# Large Scale Relationship between Aquatic Insect Traits and Climate

**DOI:** 10.1371/journal.pone.0130025

**Published:** 2015-06-16

**Authors:** Avit Kumar Bhowmik, Ralf B. Schäfer

**Affiliations:** Quantitative Landscape Ecology, Institute for Environmental Sciences, University of Koblenz-Landau, Landau in der Pfalz, Germany; Consiglio Nazionale delle Ricerche (CNR), ITALY

## Abstract

Climate is the predominant environmental driver of freshwater assemblage pattern on large spatial scales, and traits of freshwater organisms have shown considerable potential to identify impacts of climate change. Although several studies suggest traits that may indicate vulnerability to climate change, the empirical relationship between freshwater assemblage trait composition and climate has been rarely examined on large scales. We compared the responses of the assumed climate-associated traits from six grouping features to 35 bioclimatic indices (~18 km resolution) for five insect orders (Diptera, Ephemeroptera, Odonata, Plecoptera and Trichoptera), evaluated their potential for changing distribution pattern under future climate change and identified the most influential bioclimatic indices. The data comprised 782 species and 395 genera sampled in 4,752 stream sites during 2006 and 2007 in Germany (~357,000 km^²^ spatial extent). We quantified the variability and spatial autocorrelation in the traits and orders that are associated with the combined and individual bioclimatic indices. Traits of temperature preference grouping feature that are the products of several other underlying climate-associated traits, and the insect order Ephemeroptera exhibited the strongest response to the bioclimatic indices as well as the highest potential for changing distribution pattern. Regarding individual traits, insects in general and ephemeropterans preferring very cold temperature showed the highest response, and the insects preferring cold and trichopterans preferring moderate temperature showed the highest potential for changing distribution. We showed that the seasonal radiation and moisture are the most influential bioclimatic aspects, and thus changes in these aspects may affect the most responsive traits and orders and drive a change in their spatial distribution pattern. Our findings support the development of trait-based metrics to predict and detect climate-related changes of freshwater assemblages.

## Introduction

Freshwater ecosystems are among the most threatened in terms of biodiversity loss, because of overexploitation, water pollution, invasive species, flow modification and degradation of habitat [1,2]. While these are mainly local scale stressors, patterns of freshwater assemblages on large spatial scales are driven by environmental variables such as climate, geology and acid deposition [3,4]. Climate is the predominant environmental driver that directly affects the thermal and flow regimes of freshwater bodies and thus controls organismal growth and performance [5]. Moreover, climate may influence the biogeography of organisms and shape geology and acid deposition on large spatial scales [4]. Thus, quantifying the relationship between climate and large scale freshwater assemblages can help to understand and predict climate change effects on freshwater ecosystems [6].

Traits of organisms, defined as biological (life history) characteristics and ecological preferences that may evolve from a number of developmental, morphological, physiological and behavioral adaptations of organisms to their environment [7,8], have shown considerable potential as indicators of multiple stressor effects in freshwater ecosystems [9]. Traits were also shown to provide a link to important freshwater ecosystem functions and services [10,11]. Especially on large scales, trait variability is less than the taxonomic variability [12] and therefore traits are more suitable for quantifying the relationship between climate and freshwater assemblage composition.

Several biological and ecological traits of freshwater organisms have been associated with climate change in previous studies. For example, organisms that prefer cold temperature [13] and with low dispersal capacity [14] exhibited range contractions, large-bodied (>4 cm) and semivoltine organisms decreased [15], rheophil and rheobiont organisms declined or disappeared [16] and the distribution of organisms with narrow niche breadth, restricted resource distribution and short flight period shrinked [17]. Consequently, such traits were assumed to be vulnerable and employed to assess risk of individual organism groups, i.e. Ephemeroptera, Plecoptera and Trichoptera [18–20], sites (streams and lakes) and ecoregions [4,21] from climate change. For example, rheobiont and cold temperature preferring organisms were assumed to be threatened by climate change and in concert with additional traits were used to identify potentially vulnerable European ephemeropterans, plecopterans and trichopterans [18–20]. The same hypothesized climate-vulnerable traits and organism groups were used to identify the Swedish streams and lakes [21] and European eco-regions [4] that are at the highest risk of adverse climate change effects. However, the large scale relationship between the variability of freshwater assemblage trait composition and climate has rarely been quantified [22]. Quantification of the trait-climate relationship allows to identify the most vulnerable and tolerant organism groups and their traits as well as to examine whether organism groups or traits differ in their vulnerability to specific aspects of climate change, e.g. change in winter temperature or precipitation [5,13,16].

Freshwater assemblages are distributed non-randomly along spatial gradients, i.e. longitude, latitude and altitude on large scales, leading to spatial patterns in their trait composition [23]. Spatial autocorrelation, referring to the concept that organismal traits observed at a given stream site are more similar to traits in close sites than in distant sites, measures the strength of spatial pattern in the distribution of organismal traits [24]. Trait spatial autocorrelation can be endogenous, i.e. arises from ecological processes such as dispersal and reproduction, or exogenous, i.e. induced by environmental drivers like climate [23,25]. Climate shows a strongly positive autocorrelation, i.e. closer regions have a more similar climate than distant ones. The spatial patterns of freshwater organisms with climate-associated traits often reflect this spatial autocorrelation of climate. For example, a recent study on stream invertebrate taxonomic richness and composition suggested that spatial autocorrelation in organism groups with aerial dispersal ability (e.g. Ephemeroptera, Plecoptera and Trichoptera) is mainly related to large scale climate variability [24]. Moreover, organisms preferring cold temperature were shown to predominantly occur in alpine regions with high altitudes, whereas those preferring warm temperature tend to occur in lowland regions [26]. Hence, freshwater organisms with climate-associated traits that exhibit strong relationship with climate in their spatial autocorrelation are most likely to change their distribution pattern under future climate change [27]. However, little is known about the relationship between the spatial pattern in the assumed climate-associated traits and climate on large spatial scales.

We empirically quantified the large scale relationship of the German stream macroinvertebrate assemblage trait composition with climate. Our research questions were two-fold: (i) which of the climate-associated traits and organism groups show the highest response to climate and highest potential for changing distribution pattern under future climate change?, and (ii) which are the most influential climatic aspects for the traits and organism groups showing the highest response and potential for changing distribution? We selected climate-associated traits from six grouping features, i.e. four biological and two ecological grouping features (“grouping feature” and “trait” follow the unified terminologies suggested by [28]) that have been used in previous large scale studies to indicate vulnerability [4,18–21] and five orders of stream macroinvertebrates that are aerial dispersers, i.e. aquatic insects [29]. Climate was measured as 35 global bioclimatic indices (BIs) that are biologically and ecologically relevant [30] and vary considerably over Germany due to its diverse topography [31]. The large scale variability and spatial distribution pattern of the aquatic insect assemblage trait composition were quantified and checked for their relationship with the combined and individual BIs.

## Materials and Methods

### Concepts of scale

We covered two concepts of spatial scale: (i) spatial extent or size of the study area and (ii) spatial resolution or wavelength of variability of the variables [25]. Ours is a large scale study from both conceptual points of view, i.e. large extent (Germany, area approximately 357,000 km²) and large (coarse) resolution (approximately 18 km (10 arcminutes) based on the variability of the BIs). We use the terms “large scale” and “scale” for both concepts. When we refer to the scale of Germany, we mean extent; but when we refer to the scale of the relation, e.g. variability and pattern, we mean resolution. Moreover, when we refer to the scale of relationship, we refer to both concepts.

### Data and processing

#### Aquatic insect data

**Fig 1:**
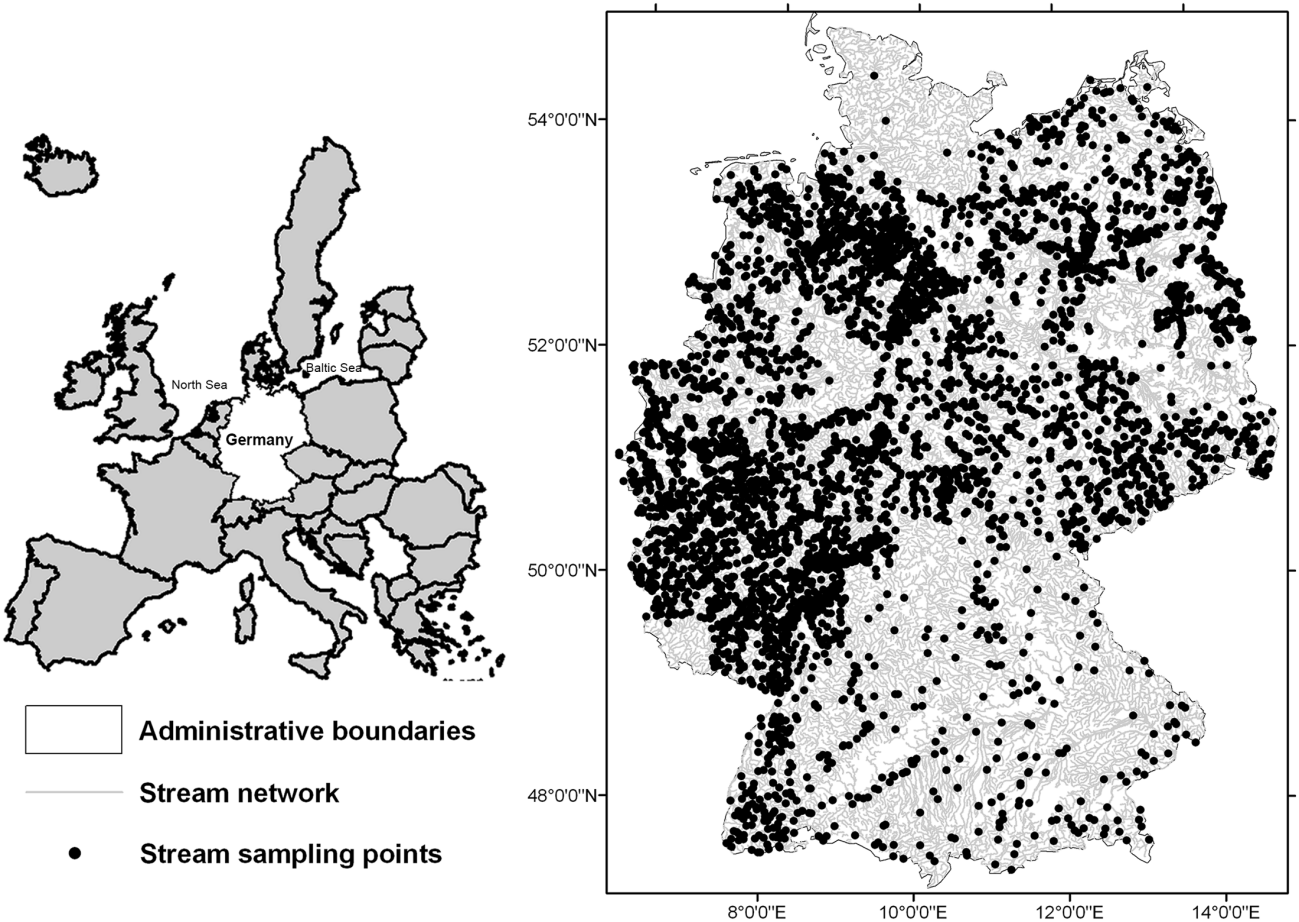
Distribution of the 4,752 stream sites sampled by the German national bio-monitoring program during 2006–2007. Spatial reference system is WGS 1984.

We used governmental biomonitoring data on macroinvertebrates from 4,752 stream sites (i.e. stream reaches with a maximum of 20 meters length) sampled during 2006–2007 that covered the whole spatial extent of Germany (Fig 1). Data coverage in terms of number of sites was lower (approximately 1% of the total number of sites) in the southeast and northwest (in proximity to the North and Baltic sea) than in the other regions. The biomonitoring data was produced following a standardized protocol, where a pooled sample was taken from all major habitat types in a stream site [32]. Samples were collected from the middle and small sized streams in each ecoregion of Germany. For more details on biological sampling, subsampling and sorting see AQEM CONSORTIUM, Rolauffs et al. and Biss et al. [32–34]. Given the semi-quantitative nature of macroinvertebrate data, the data were originally reported as abundance classes where the classes approximated log-transformed abundance data following the classes of the saprobic index (for the description of the abundance classes see AQEM CONSORTIUM and Rolauffs et al. [32,33]). Thus, the abundance classes varied on a scale of zero to seven; zero meaning no abundance, i.e. absence and seven the highest abundance [33]. Overall, abundance classes for 2,099 stream macroinvertebrates were available. Abundance data were preferred over presence-absence data because more powerful hypothesis tests are available for abundance data in spatial pattern analysis of assemblage compositions and studies of turnover rates [35].

We examined the homogeneity of taxonomic resolution and found that the organisms were reported at different taxonomic levels (from class to species). We took the subset of 1,901 organisms (91%) that were reported at genus (660) and species (1,241) levels. From this subset, we selected the aquatic insect orders, namely Diptera (True flies), Ephemeroptera (Mayflies), Odonata (Dragonflies and Damselflies), Plecoptera (Stoneflies) and Trichoptera (Caddisflies) (Table 1). Aerial dispersers are more suitable for large scale analyses than exclusive aquatic dispersers, because they can disperse through the landscape and are not limited to the stream network [24,29]. Moreover, these orders were also used in previous large scale studies to indicate climate vulnerability [4,18–21] and information for the selected traits were available for all organisms in these orders. This resulted in 782 species [and 395 genera] that comprised 384 (216) dipterans, 101 (39) ephemeropterans, 42 (33) odonates, 52 (36) plecopterans and 203 (71) trichopterans. Next, in case that a taxon was identified at genus level for more than 1% of stream sites, we converted all species belonging to this genus to genus level. This was the case for 73% of the species and was done to avoid artifacts from potential spatial pattern linked to the taxonomic resolutions, for instance mainly genus level identification in regions with low data coverage.

**Table 1:** Explained variances and spatial autocorrelation in the traits of each order and full data by the bioclimatic indices.

Grouping features and traits	Explained variability (%)						Explained spatial autocorrelation (%)					
	Diptera	Ephemeroptera	Odonata	Plecoptera	Trichoptera	Full data	Diptera	Ephemeroptera	Odonata	Plecoptera	Trichoptera	Full data
**Biological traits**												
*Dispersal capacity* ^a^												
Unknown	NA	NA	NA	NA	4.7	4.1	NA	NA	NA	NA	44	25
Low	NA	*	NA	*	12	16	NA	*	NA	*	38	68
High	NA	NA	NA	NA	10	15	NA	NA	NA	NA	54	38
*Average*	*NA*	*NA*	*NA*	*NA*	*9*.*1*	*12*	*NA*	*NA*	*NA*	*NA*	*45*	*44*
*Maximal body size* ^b^												
> 0.25 cm to 0.5 cm	8.0	10	NA	32	19	17	1.0	35	NA	79	51	78
> 0.5 cm to 1 cm	14	13	NA	9.7	11	12	62	61	NA	83	50	65
> 1 cm to 2 cm	10	12	18	21	14	18	41	35	32	49	60	75
> 2 cm to 4 cm	16	12	10	8.6	12	14	39	80	88	26	26	65
> 4 cm to 8 cm	13	NA	16	NA	NA	13	3.0	NA	11	NA	NA	46
*Average*	*12*	*12*	*15*	*18*	*14*	*15*	*29*	*53*	*44*	*59*	*47*	*66*
*Reproductive capacity* ^a^												
Flexible	NA	23	NA	25	4.1	10	NA	66	NA	90	65	37
Semivoltine	11	10	NA	7.0	22	14	55	29	NA	42	52	47
Univoltine	16	9.3	NA	14	28	8.6	53	70	NA	89	68	60
Bivoltine	12	11	NA	NA	15	20	35	70	NA	NA	11	71
Trivoltine	7.6	25	NA	NA	NA	9.7	2.3	60	NA	NA	NA	34
Multivoltine	17	15	NA	NA	3.3	9.1	76	41	NA	NA	36	16
*Average*	*13*	*15*	*NA*	*15*	*14*	*12*	*44*	*56*	*NA*	*74*	*46*	*44*
*Resistance to drought* ^a^												
Unknown resistance type	NA	NA	NA	18	8.2	10	NA	NA	NA	3.7	92	51
No drought resilience	NA	NA	NA	NA	13	7.9	NA	NA	NA	NA	28	0.1
Egg diapause	NA	17	NA	18	NA	41	NA	61	NA	46	NA	73
Larvae diapause	NA	16	NA	NA	6.1	13	NA	67	NA	NA	60	66
Adult diapause	NA	NA	NA	NA	11	13	NA	NA	NA	NA	18	15
*Average*	*NA*	*16*	*NA*	*18*	*9*.*8*	*17*	*NA*	*64*	*NA*	*25*	*49*	*41*
**Ecological traits**												
*Current preference* ^a^												
Indifferent	9.2	NA	NA	18	5.3	24	33	NA	NA	63	2.7	67
Limnobiont	6.3	NA	9.8	NA	22	15	22	NA	42	NA	65	61
Limnophil	7.1	25	6.5	27	43	41	90	56	5.6	63	75	77
Limno to Rheophil	5.1	4.4	12	8.7	15	8.1	12	42	13	36	57	74
Rheo to Limnophil	12	15	14	7.3	6.7	13	52	58	63	13	82	71
Rheophil	27	17	20	34	26	40	69	58	67	72	68	85
Rheobiont	21	7.3	8.7	13	22	30	52	13	89	65	54	59
*Average*	*13*	*14*	*12*	*18*	*20*	*25*	*47*	*45*	*47*	*52*	*58*	*71*
*Temperature preference* ^a^												
Eurytherm	26	21	NA	22	14	34	84	65	NA	67	66	87
Very cold	8.9	34^#^	NA	16	17	50^#^	83	76	NA	34	59	82
Cold	30	14	NA	8.5	19	42	71	84	NA	65	69	91^#^
Moderate	15	33	NA	6.9	8.5	8.6	84	88	NA	67	98^#^	60
Warm	24	15	NA	10	15	24	82	80	NA	16	66	86
*Average*	*21*	*23*	*NA*	*13*	*15*	*32* ^#^	*81*	*79*	*NA*	*50*	*71*	*81* ^#^
***Average over traits and orders***	***14***	***16*** ^***#***^	***13***	***16*** ^***#***^	***15***	***19***	***50***	***59*** ^***#***^	***46***	***53***	***54***	***59***

The detailed information on the traits including the source databases and their occurrence and variability in Germany are presented.

^a^data source: freshwater ecology database (www.freshwaterecology.info) (Schmidt-Kloiber & Hering, 2012)

^b^data source: Tachet database (Usseglio-Polatera *et al*., 2000)

^NA^ Trait not occurring

* Trait omitted from the analysis because of zero variability (i.e. all organisms have same trait) and therefore the abundance weighted trait cannot be computed

^#^ Traits and orders showing the strongest relationship with the bioclimatic indices in their variability and spatial autocorrelation

#### Biological and ecological traits data

Biological and ecological traits of aquatic insects were taken from two databases: (i) the freshwater ecology database (www.freshwaterecology.info) [36] and (ii) the Tachet database [37]. The trait information is recorded at species level in the freshwater ecology database, whereas they are recorded mostly at genus and species levels in the Tachet database. In both databases, the membership state (see Schmera et al. [28] for terminology) of a taxon for a particular trait is generally described on a scale from zero to 10 (with exceptions for the Tachet database); zero indicates no membership and 10 the highest membership state. We selected the climate-associated traits from six grouping features (for details see Table 1) and converted the membership state of the traits into percentages as suggested by Schmera et al. [28]. These traits were selected because they were used in previous large scale studies to indicate vulnerability [4,18–21] and have the highest data coverage for the macroinvertebrates in German streams. We also compared the membership states of the insect orders for each of the selected traits (Table A in S2 File).

#### Calculation of assemblage trait composition

**Fig 2:**
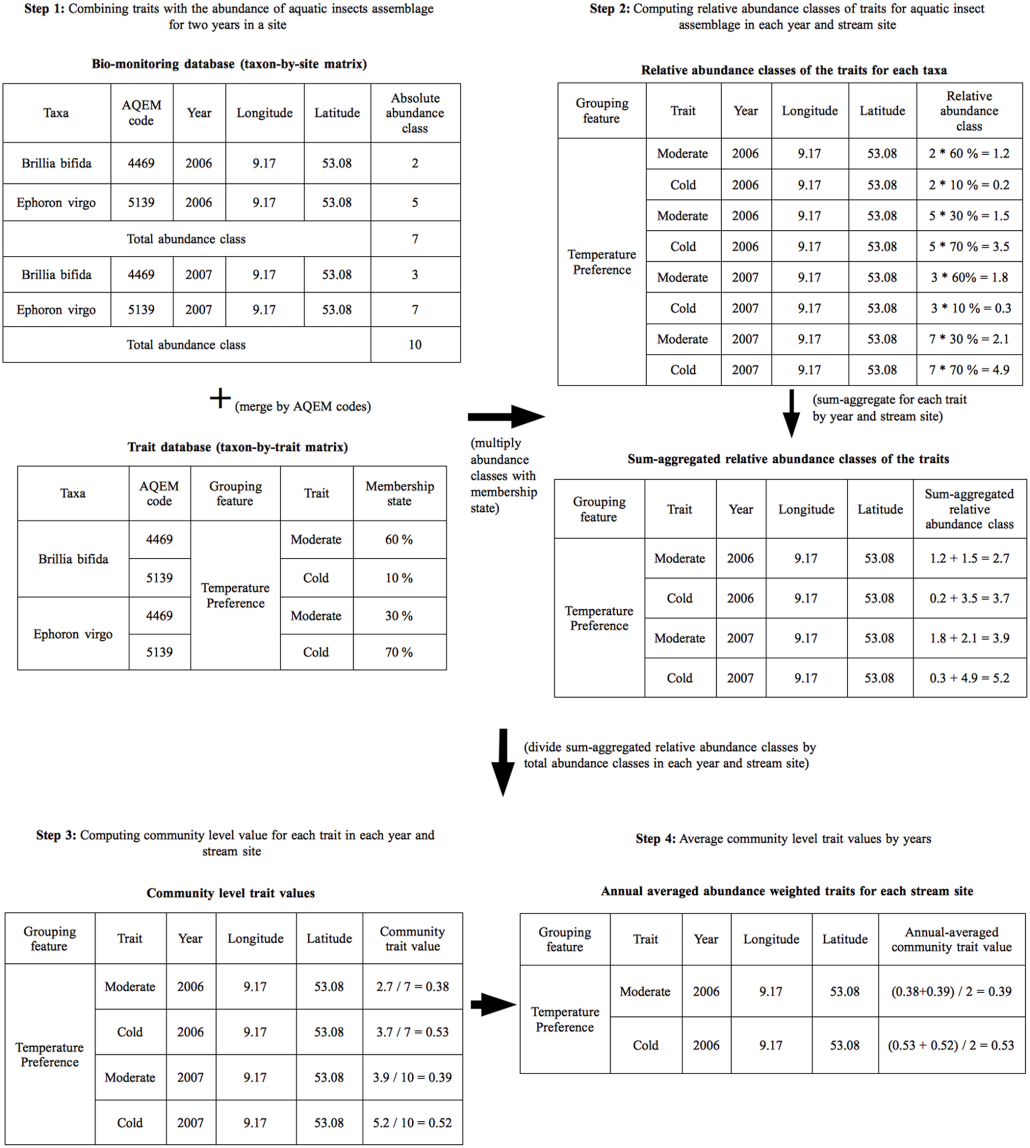
Conversion steps from abundance classes of the selected aquatic insects to trait compositional (annual averaged abundance weighted traits) data.

**Fig 3:**
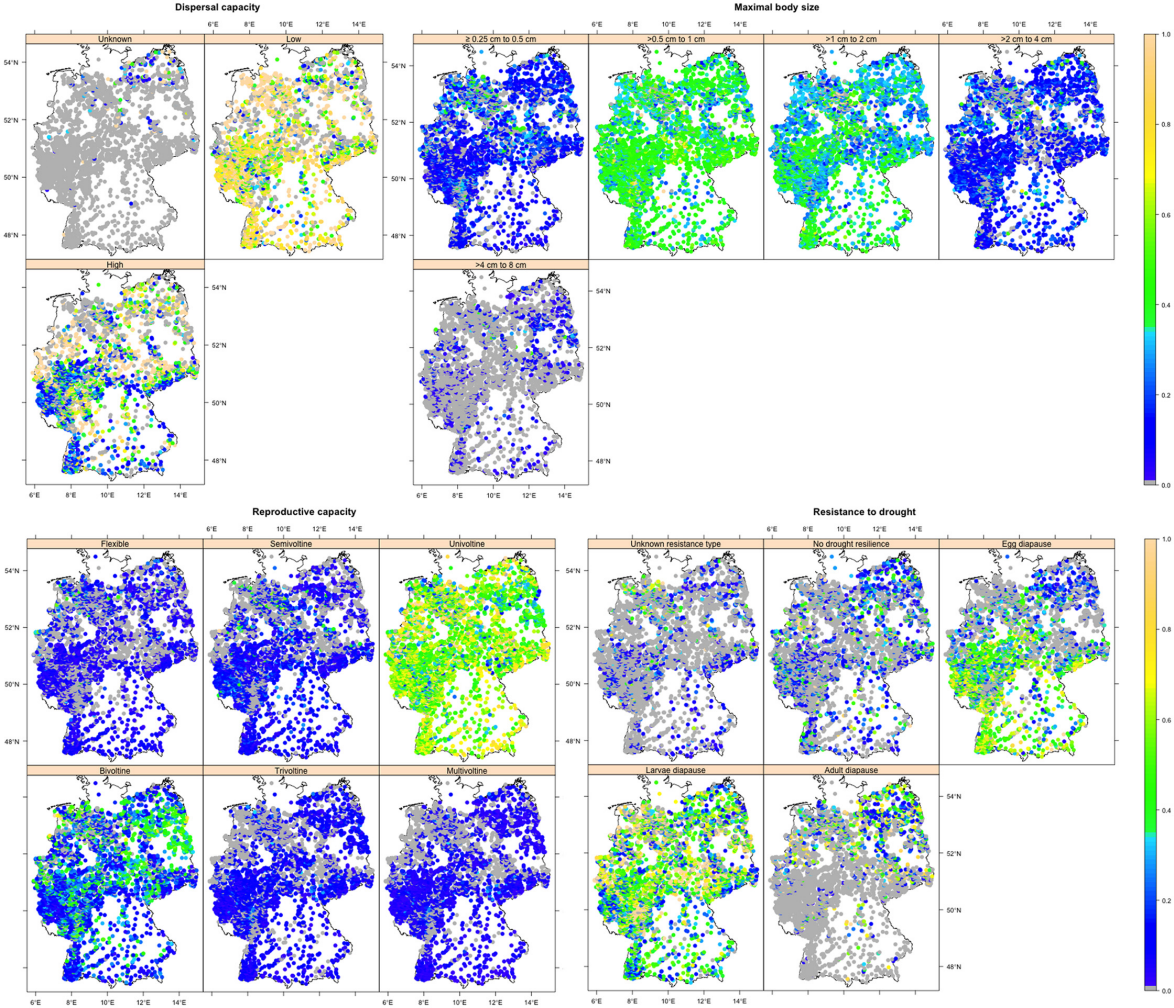
Annual averaged abundance weighted traits across 4,752 stream sites in Germany for the biological traits of the full data. The figure sub-captions and panel captions indicate names of grouping features and traits, respectively. The gray dots indicate the zero abundance, i.e. trait absence.

**Fig 4:**
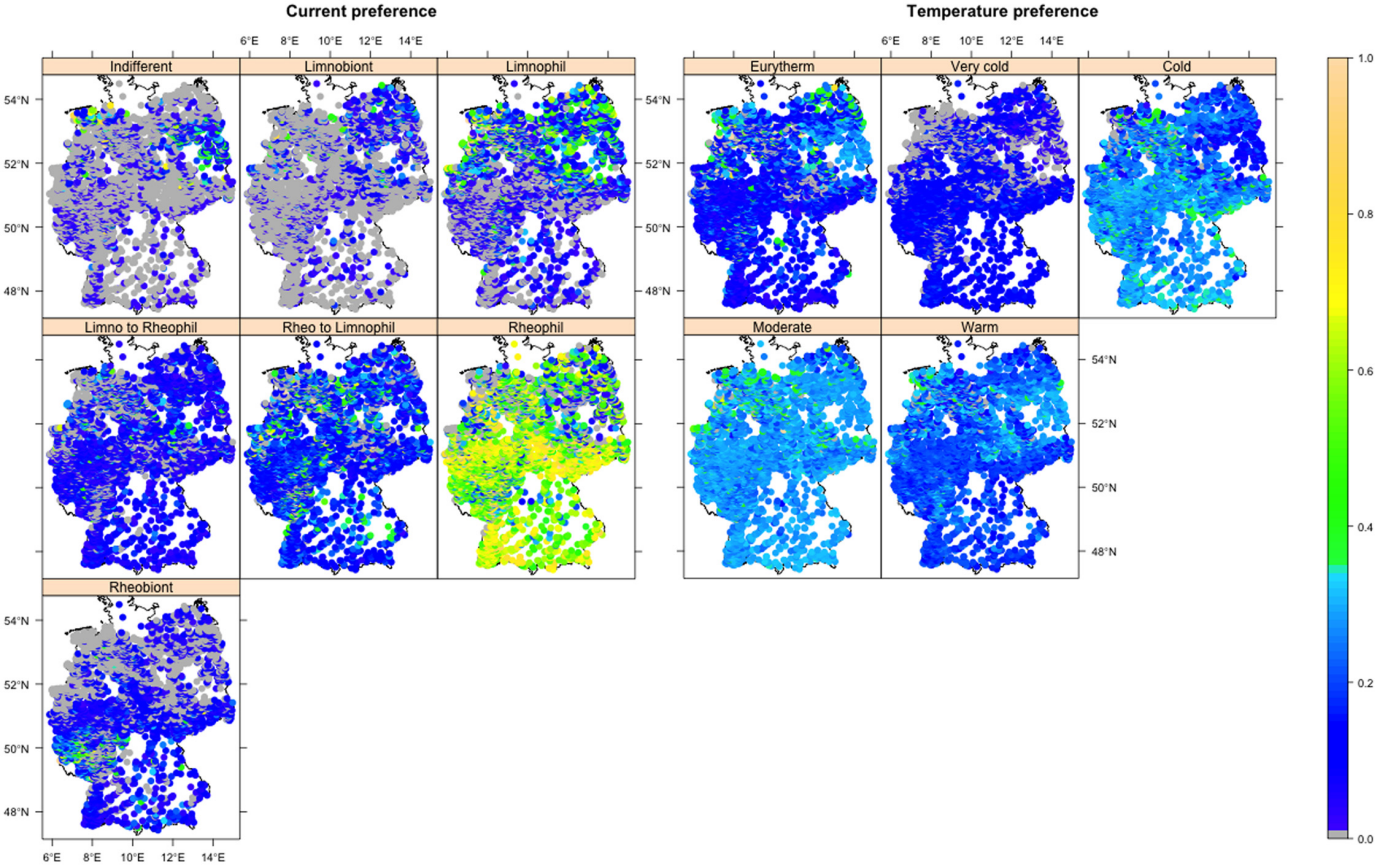
Annual averaged abundance weighted traits across 4,752 stream sites in Germany for the ecological traits of the full data. The figure sub-captions and panel captions indicate names of grouping features and traits, respectively. The gray dots indicate the zero abundance, i.e. trait absence.

The biomonitoring data were linked to the trait data using the codes of “The development and testing of an integrated assessment system for the ecological quality of streams and rivers throughout Europe using benthic macroinvertebrates” (AQEM) project to avoid discrepancies in naming conventions [38]. Each of the species was assigned with the traits using their corresponding percentage membership states that were multiplied with the absolute abundance classes of the species for a site to compute relative abundance classes for the traits (Fig 2). To assign trait information to genera, we calculated the median of the related species level information following Schmidt-Kloiber and Nijboer [39] except for maximal body size where genus level information were available in the Tachet database for all genera. Subsequently, the assemblage trait composition, i.e. abundance weighted trait (AWT) was calculated following the procedure described in [40] and as outlined in Fig 2. The AWT was calculated as a measure of assemblage trait composition because it is the most frequently used metric to assess the relationship between assemblage traits and environmental variables [41,42]. Note that we use the term assemblage trait composition to improve readability, although the assemblage data was restricted to aquatic insects, and hence does not represent the complete macroinvertebrate assemblage. The calculation resulted in annual averaged abundance-weighted traits (AWT) for each insect order (Fig A in S1 File) and for the combined (full) data (Figs 3 and 4) for each stream site. The calculation was omitted for the dispersal capacity of ephemeropterans and plecopterans because the grouping feature consisted of only one trait (low dispersal). However, they were included in the calculation for the full data.

#### Bioclimatic indices and altitude data

The 35 bioclimatic indices (BI, denoted as “Bio01” to “Bio35”, see Table 2 for details) for temperature, precipitation, radiation and moisture were collected from the global climatologies for bioclimatic modeling (CliMond) database (www.climond.org) [30]. A previous study showed that these BIs can provide an approximation of climate impact on assemblage patterns, despite the omission of confounding endogenous factors such as biotic interactions, evolutionary change and dispersal potential [43]. The scale of variability was determined by the spatial resolution of the BI raster, which is 10 arc-minutes (approximately 18 km). The digital elevation model (giving altitude over mean sea level) for Germany was collected from the ASTER GDEM on one arc-second (approximately 30 m) resolution [44]. The altitude raster was resampled to the resolution of the BI rasters to extract altitude information for each BI raster cell.

**Table 2:** Explained variances and spatial autocorrelation by the individual bioclimatic indices in the traits and orders with the highest climate response and potential for changing distribution pattern.

Variable Number	Variables (unit)	Explained variance (%)		Explained spatial autocorrelation (%)	
		Very cold temperature preferring insects	Very cold temperature preferring Ephemeroptera	Cold temperature preferring insects	Moderate temperature preferring Trichoptera
Bio01	Annual mean temperature (°C)	8.6	9.1	3.2	61
Bio02	Mean diurnal temperature range (°C)	3.2	1.6	24	57
Bio03	Isothermality	7.2	2.9	26	50
Bio04	Temperature seasonality	2.8	0.9	5.9	61
Bio05	Max temperature of warmest week (°C)	6.1	5.8	11	63
Bio06	Min temperature of coldest week (°C)	1.8	3.5	6.1	59
Bio07	Temperature annual range (°C)	0.5	0.1	6.6	61
Bio08	Mean temperature of wettest quarter (°C)	5.3	4.2	3.1	59
Bio09	Mean temperature of driest quarter (°C)	0.2	0.7	11	64
Bio10	Mean temperature of warmest quarter (°C)	13	12	8.2	59
Bio11	Mean temperature of coldest quarter (°C)	1.5	3.4	9.7	63
Bio12	Annual precipitation (mm)	15	13	29	37
Bio13	Precipitation of wettest week (mm)	13	11	31	36
Bio14	Precipitation of driest week (mm)	18^#^	14	32	33
Bio15	Precipitation seasonality	1.4	0.5	5.3	63
Bio16	Precipitation of wettest quarter (mm)	13	11	28	41
Bio17	Precipitation of driest quarter (mm)	15	12	27	40
Bio18	Precipitation of warmest quarter (mm)	12	11	25	45
Bio19	Precipitation of coldest quarter (mm)	12	10	18	54
Bio20	Annual mean radiation (W m-2)	3.7	2.5	7.8	60
Bio21	Highest weekly radiation (W m-2)	2.3	1.2	2.2	58
Bio22	Lowest weekly radiation (W m-2)	12	8.0	33	49
Bio23	Radiation seasonality	17	11	46^#^	43
Bio24	Radiation of wettest quarter (W m-2)	1.8	1.3	4.4	60
Bio25	Radiation of driest quarter (W m-2)	1.7	1.5	2.9	65^#^
Bio26	Radiation of warmest quarter (W m-2)	0.1	0.1	5.4	63
Bio27	Radiation of coldest quarter (W m-2)	12	8.6	28	54
Bio28	Annual mean moisture index	16	14^#^	18	46
Bio29	Highest weekly moisture index	8.2	8.1	5.4	58
Bio30	Lowest weekly moisture index	14	13	18	48
Bio31	Moisture index seasonality	16	13	21	43
Bio32	Mean moisture index of wettest quarter	9.8	9.2	7.5	60
Bio33	Mean moisture index of driest quarter	14	13	20	47
Bio34	Mean moisture index of warmest quarter	15	13	21	45
Bio35	Mean moisture index of coldest quarter	11	10	11	55

Details on the bioclimatic variables are extracted from Kriticos et al. **[30]** and https://www.climond.org/Resources.aspx.

^#^ the highest explained variability and spatial autocorrelation in a trait of insects or an order by a bioclimatic index

Although no clear gradient in the BIs was found for Germany, lower temperatures and higher precipitation were mostly observed in the southern regions, whereas higher temperatures and lower precipitation were mostly observed in the northern regions (Fig B in S1 File). For example, observed ranges of the annual mean temperature (Bio01) and annual precipitation (Bio12) are 2 to 5°C and 7 to 12°C, and 1200 to 1600 mm and 600 to 800 mm in the southern and northern regions, respectively. The northern and southern regions are portrayed as flat (zero to 250 m above sea level) and mountainous (600 to 1800 m above sea level), respectively (Fig C in S1 File). The BIs showed significant (p < 0.001) spatial autocorrelation, i.e. average Moran's I = 0.28 (Table B in S2 File). Significant spatial gradients were also observed for the BIs (Table B in S2 File). Longitudinal (North—South) and altitudinal (high—low) gradients were both stronger than the latitudinal gradient (East—West) for most of the BIs. Longitude and altitude of the BI cells showed significantly high correlation (r = -0.8, p < 0.001) with each other and thus indicates that the dominant climatic variation along the North—South (longitudinal) gradient on the scale of Germany (also observed in Fig B in S1 File) may be attributed to topography, i.e. altitude (low—high).

#### Pre-processing of BI and AWT data

The stream sites covered 72% of the total BI raster cells within the boundary of Germany (Fig D in S1 File). However, given the relatively coarse resolution of the BI data, multiple sites were often located in one BI raster cell. Therefore, we aggregated the AWTs in all sites within a BI raster cell via averaging and assigned the result to that cell to avoid pseudo replication. The BIs exhibited considerable multicollinearity (Fig E in S1 File) and therefore we conducted a principal component analysis (PCA) to arrive at independent variables and extracted the scores of the 35 orthogonal principal components, as suggested by Graham [45], for latter analysis. PCA was preferred over residual and sequential regression as this also obliterates the likely effects of the latent spatial variables (as described above) on the BIs [45]. All data processing and PCA of BIs were done in R software environment [46] using the packages “sp” [47], “vegan” [48], “raster” [49] and “maptools” [50].

### Analyses of the spatial relationship between traits and climate

The spatial relationship between the aggregated AWT per BI cell and the BIs was analyzed in four steps (Fig F in S1 File). First, we checked for spatial autocorrelations in the AWT (Table C in S2 File). The spatial autocorrelation was analyzed using Global Moran's I (see Bonada et al. [24] for details on computation), where great circle distances among BI cell pairs were given as weights based on the simplified assumption that the selected species disperse symmetrically during their terrestrial life stage [29]. The computations of spatial autocorrelations were done using the R package “ape” [51].

Second, zero-or-one inflated beta regression models were fitted with the AWT as response and 35 principal component scores of the BIs as explanatory variables [52]. This was done to identify the traits and insect orders with the highest climate response. We used zero-or-one inflated beta regression because the response variables were proportional data and included many zeros and ones [53]. The models were fitted for the AWT of each order and the full data and the adjusted R^2^s were calculated to identify the explained variance by the BIs. The zero-or-one-inflated beta regression model fitting was done using the R package “gamlss” [54].

In the third step, we checked for spatial autocorrelation in the residuals of the trait-climate models using Moran's I as outlined above. The Moran's I values for the residuals of the trait-climate models were subtracted from the complete Moran's I values for the AWT (computed at the first step). Thus, the percentage of trait spatial autocorrelation that is associated with the BIs was identified. This was done to identify the traits and orders that show the highest potential for changing their distributional pattern, i.e. redistribution under future climate change.

In a final step, the zero-or-one inflated beta regression models were re-fitted with the AWT for the previously identified traits and orders with the highest climate response and potential for redistribution as response variables and 35 BIs (original values) separately as explanatory variables. The BIs with the highest explanatory power in terms of R^2^ were identified for the traits and insect orders with the highest climate response. To identify the BIs explaining the highest amount of spatial autocorrelation in the traits and in the insect orders with the highest potential for redistribution, we computed the Moran's I in the residuals of the trait-individual BI models and subtracted them from the complete Moran's I computed at the first step.

## Results and Discussion

### Which of the climate-associated traits and organism groups show the highest response to climate and highest potential for changing distribution pattern under future climate change?

We quantified the amount of large scale variability and spatial autocorrelation in the assumed climate-associated traits from six grouping features and five aquatic insect orders of the freshwater assemblages that is explained by 35 global BIs. The BIs explained 19% of the large scale variability in the AWT of the full data on average (Table 1). Traits of the temperature preference grouping feature were the most responsive (32% on average) to the BIs, and the insects with very cold temperature preference (50%) showed the highest response. Among the insect orders, Ephemeroptera and Plecoptera (16%) showed the highest response to the BIs on average, and the ephemeropterans with very cold temperature preference (33%) showed the highest response in particular (Table 1).

The highest response of the traits of the temperature preference grouping feature, particularly of the very cold and cold preference may be due to traits of temperature preference grouping feature being the product of several underlying climate-associated biological traits [19,55,56]. For example, cold temperature preference of the selected aquatic insects in our study was significantly related to low dispersal capacity, large body size (>4 cm), low reproductive capacity (semivoltine) and resistance to drought (egg diapause) (Table D in S2 File), and together they explained 55% of the variability in cold temperature preference. Likewise, warm temperature preference of the insects was related to high dispersal capacity, small body size (≤0.5 cm), high reproductive capacity (multivoltine) and resistance to drought (adult diapause) (Table D in S2 File), and together they explained 48% of the variability in warm temperature preference. These findings are in agreement with other studies on the association of traits with climate change. For example, insects with low dispersal are often characterized by a restricted temperature (cold) niche and hence are more affected by change in temperature regimes, e.g. contractions of alpine regions than the insects with high dispersal ability [18–20,57]. Large-bodied insects generally lack efficient respiration and thus have high ectotherm oxygen demand and hence typically inhabit streams with high oxygen supply, i.e. cold water streams [15,58,59]. Hence, we argue that the highest response of the traits of temperature preference grouping feature to the BIs in our study rather follows from the response of several underlying climate-biological traits relationships. Thus, we envisage an adverse effect of global warming on the insects inhabiting cold water streams in Germany because their biological and ecological niche will be contracted. This prediction is in line with Poff et al. [5], where temperature has been shown to be mostly accountable for the differences in the sensitivity of stream macroinvertebrate traits across geographic space and also with Lawrence et al. and Stamp et al. [15,56] where major declines in macroinvertebrates that inhabit cold water streams were reported as a result of climate change.

The differences in the response of insect orders observed in our study are related to their biological and ecological traits (Tables A and D in S2 File) [4,18–20,58]. Although European ephemeropterans were found to be generally tolerant to climate change [4], we observed the highest BI response in the German ephemeropterans with very cold temperature preference (Table 1). This indicates that ephemeropterans inhabiting very cold water streams in Germany are also vulnerable to climate change because of shrinking ecological niche [60]. Plecopterans showed equally high response as ephemeropterans because they show high membership state for the very cold and cold preference traits, which showed the highest response to the BIs (Table 1 and Table A in S2 File). Generally, plecopterans have a very narrow environmental tolerance with nymphs living mainly in cold and well-oxygenated running water and adults showing low flight ability [18,59]. Hence, plecopterans have never transitioned to thermally variable lentic water and are thus vulnerable to increasing temperature and severe drought episodes [58]. Thus, we also anticipate an adverse effect of climate change on plecopterans in Germany. Overall, our results indicate that insects with traits such as preference for cold water (due to several underlying traits), and from certain orders, i.e. Ephemeroptera and Plecoptera may indeed be more vulnerable to climate change than others (Table 1). Thus, we suggest that future studies on the vulnerability of macroinvertebrate assemblage traits to climate change should particularly focus on traits and orders exhibiting the strongest signal to climate.

Regarding the potential for changing distribution pattern, i.e. redistribution, on average, 59% of the spatial autocorrelation in the AWT of the full data was associated with the BIs (Table 1). The BIs explained the highest spatial autocorrelation in the temperature preference (81%), particularly in the insects with cold temperature preference (91%) (Table 1). More than 50% of the spatial autocorrelation for the majority (62%) of the traits in the insect orders was associated with the BIs. The BIs explained the highest amount of spatial autocorrelation for the insect order Ephemeroptera (59%) in general, and for the Trichoptera with moderate temperature preference (97%). The amount of large scale variability explained by the BIs (described above) in insect traits and orders showed positive significant correlation (r = 0.5, p < 0.001) with the amount of explained spatial autocorrelation. This indicates that the traits and orders showing higher response to the BIs also exhibit a higher potential for changing spatial distribution pattern under changing BIs and vice-versa. Overall, the spatial distribution pattern, i.e. patchiness in the aquatic insects on large scales mostly originate from their high response to spatially autocorrelated climate that is line with Bonada et al. and Domisch et al. [24,26].

The highest potential for redistribution in the traits of temperature preference grouping feature and insect order Ephemeroptera, and trichopterans preferring moderate temperature also presumably relates to their strong covariation with underlying climate-associated biological and ecological traits as discussed above (Tables A and D in S2 File). For example, trichopterans showed high membership state for the underlying biological traits of the moderate temperature preference, i.e. small body size (< 0.5 cm) and high drought resistance (adult diapause) (Tables A and D in S2 File), and hence moderate temperature preferring trichopterans showed the highest potential for redistribution. The redistribution of traits and orders may occur through local extinction of vulnerable insects and thus range contraction [19], or by expansion of the range of tolerant macroinvertebrates in response to climate change [61,62]. Moreover, given that there is a strong association of the spatial distribution pattern of AWT of the insect orders individually (Fig A in S1 File) and of the full data (Figs 3 and 4) with the longitudinal gradient (which is coherent with the observed longitudinal spatial distribution pattern in the climate sensitive European stream macroinvertebrates [4,19,20]), and the BIs also showed a major longitudinal gradient with high correlation to altitude (Fig B in S1 File and Table B in S2 File), the redistribution may occur along the longitudinal (altitudinal) gradient. For example, a higher proportion of insects (0.4) and ephemeropterans (0.3) with cold temperature preference were observed in the cooler southern mountainous regions than in the warmer flat North of Germany (Fig 4 and Fig A in S1 File) that may shrink their distribution range. By contrast, trichopterans with moderate temperature preference that predominantly (0.5) occur in the warmer flat northern regions than in the cooler South may extend their range from North to South because more streams will be suitable for their habitat due to increasing temperature. A similar phenomenon was observed in Hering et al. [19] where most of the European trichopterans were suggested to benefit from increasing stream temperature (78%) and decreasing current (77%). Overall, climate change may alter the trait distribution pattern especially with respect to temperature preference and for the insect order Ephemeroptera, Plecoptera, and for trichopterans with moderate temperature preference in Germany, though adaptations may occur and ameliorate the ecological effects.

The explained variability and spatial autocorrelation for the traits and orders by the BIs in our study are similar (with a few exceptions) to previous studies using aerial and exclusive aquatic dispersers on comparable spatial scales [5,24]. A study dealing with the Mediterranean basin found that climate and environmental variables together explained < 19% variability for the same insect orders (except Diptera) [24]. Moreover, a lower percentage (< 30%) of spatial autocorrelation was associated with climate and other environmental variables than in our study, and in many cases significant spatial autocorrelation remained in the residuals. This discrepancy may be explained by the fact that the study considered only two climate variables (average precipitation and temperature) whereas we considered 35 BIs. The 35 BIs used in our study better captured the climate gradient in Germany and consequently are associated with higher variability and spatial autocorrelation in the AWTM. The use of different biological endpoints, i.e. taxonomic richness in [24] and trait abundance in our study may also explain this discrepancy. In another study on the catchment scale, climate and hydrological variables together explained a similar (19%) trait variability [5] although this study was conducted on a largely different set of traits of macroinvertebrates. Overall, the differences between the studies presumably relate to the traits, organism groups and the number (dimension) of climate variables used as input in models [27].

The inclusion of other environmental drivers such as geology and stream size may decrease the amount of trait variability and spatial autocorrelation that can be attributed to the BIs, especially if drivers exhibit collinearity with the BIs. Nevertheless, other environmental drivers explained much lower taxonomic and trait variation than climate in previous studies [5,24]. Moreover, in our study, the BIs explained more than half of the spatial autocorrelation for the majority of traits, and no statistically significant (all p ≥ 0.08) spatial autocorrelation was observed in the residuals of the trait-climate models (Table 1). This indicates that the remaining trait variability and spatial autocorrelation that can be explained by other environmental drivers are either statistically insignificant or have already been captured by climate, and thus these drivers are of lower importance for the traits under scrutiny [27].

The results may bear some uncertainty regarding the northwestern and southeastern regions of Germany, which were represented by a relatively lower number of stream sites and in turn a lower coverage of BI raster cells than other regions (Fig 1 and Fig D in S2 File). However, previous studies on comparable spatial scales successfully captured macroinvertebrate trait and taxonomic variabilities and their relationships with climate and other environmental drivers, despite relying on less stream sites (lower density) [5,12,24]. Thus, we suggest that our results are sufficiently robust on the scale of Germany, though more stream sites may be required for smaller scale studies in some regions.

### Which of the climatic aspects show the strongest relationship with the traits and organism groups showing the highest response and potential for redistribution?

The explained variance and spatial autocorrelation in the most responsive traits and orders by individual BIs was on average 50% lower than by the combined BIs (Table 2). The BIs precipitation of the driest week (18%) and radiation seasonality (17%) exhibited the strongest relationship with insects preferring very cold temperature (Table 2). Precipitation and moisture indices, i.e. annual moisture index and precipitation of the driest week (both 14%), and moisture seasonality, moisture of the wettest and driest quarter (all 13%) explained the highest variance in the very cold preferring ephemeropterans. The radiation seasonality (46%), and radiation (65%) and mean temperature (64%) of the driest quarter explained the highest amount of spatial autocorrelation in the cold temperature preferring insects and moderate temperature preferring trichopterans, respectively (Table 2). Overall, these results suggest that aquatic insects in Germany may mainly be affected in response to potential changes in seasonal radiation and moisture.

In the coming decades, the winter and summer temperatures are highly likely to increase, with the strongest increase predicted for the South of Germany [60]. Moreover, winter precipitation has been predicted to increase with a larger increase in the North. By contrast, summer precipitation has been predicted to decrease in Germany with the strongest decrease in the South [60]. Thus, we anticipate an increase in winter radiation and decrease in summer moisture for the South of Germany where the majority of very cold and cold temperature preferring insects occur (Fig 4), particularly the very cold and cold temperature preferring ephemeropterans and plecopterans (Fig A in S1 File). Thus the increasing winter radiation and decreasing summer moisture may drive climate change effects on insects in general and ephemeropterans and plecopterans in particular that prefer cold water streams in Germany, and may eventually shrink their distribution range. These findings are in line with [13,15], where cold preferring stream macroinvertebrates were shown to be the most adversely affected by increasing winter temperature and decreasing summer precipitation. However, insects may also adapt to increasing temperature and decreasing precipitation [7,8]. For example, adaptations such as decreasing body size [61] and color lightening of adults [62] have been observed in insects. Trichopterans with moderate temperature living in the flat North of Germany (Fig 4 and Fig A in S1 File) may benefit from increasing radiation and recolonize upstream [19], and thus extend their distribution range from the North to the South. Overall, we anticipate a substantial change in the aquatic insect distribution pattern along the longitudinal gradient in Germany because of increasing seasonal radiation and decreasing moisture, especially in ephemeropterans and plecopterans with very cold and cold temperature preference and trichopterans with moderate temperature preference.

## Concluding remarks

The relationship of the aquatic insect assemblage trait composition with climate identified in our study can contribute to the development of trait-based metrics for predicting climate-related assemblage changes [10,11]. For example, insights from the relationship between the traits and climate could help to predict their responses to seasonal discharge, torrential floods and droughts [12]. Such insights will also support freshwater management with respect to global climate change, i.e. bio-monitoring based on climate priority traits.

## Supporting Information

S1 File Supporting Figures. Annual averaged abundance weighted traits across 4,752 stream sites in Germany for each order. The figure captions, sub-captions and panel captions indicate the names of orders, grouping features and traits, respectively. The gray dots indicate zero abundance, i.e. trait absence (**Fig A**). Extracted 35 global bioclimatic indices within the border of Germany. The indices are grouped according to their value ranges and units (°C, mm, W m^-2^ and no unit). The panel captions indicate the IDs of the indices (Bio_ID). Details on the indices and their IDs and units can be found in Table 2 and https://www.climond.org/Resources.aspx (**Fig B**). Altitudes from the mean sea level (m) within the border of Germany. Details can be found in http://asterweb.jpl.nasa.gov/gdem.asp (**Fig C**). Bioclimatic indices (BIs) raster cells that are covered (72%) by the bio-monitoring steam sites (**Fig D**). Observed multicollinearity among the 35 bioclimatic indices (BIs). Statistically significant (p<0.001) pairwise correlation coefficients (Pearson) are reported with scatterplots and histograms showing distribution. Details on the indices and their IDs and units can be found in Table 2 and https://www.climond.org/Resources.aspx (**Fig E**). Steps of the trait-climate spatial relationship analysis (**Fig F**).
(PDF)

S2 File Supporting Tables. Membership states of the five insect orders (%) for the traits of each grouping feature. The membership state (%) of an order for a trait was computed as the median of the membership states of all taxa in that order for that trait. The membership states were then scaled by the total of the membership states of an order for the traits of a grouping feature so that the membership states sum to 100% for each grouping feature (**Table A**). Spatial autocorrelations (Moran's I values) and gradients (Pearson correlations with longitude, latitude and altitude) for the bioclimatic indices (BIs) extracted at the stream sites. The Moran's I values and Pearson correlation coefficients are statistically significant at p<0.001. Details on the indices and their IDs and units can be found in Table 2 and https://www.climond.org/Resources.aspx (**Table B**). Spatial autocorrelations (global Moran's I) for abundance weighted traits in each stream macroinvertebrate order and in the full data. Observed Moran's I values are statistically significant at p<0.001 (**Table C**). Relationship between the traits of temperature preference grouping feature and the traits of remaining grouping features in terms of explained variance (%). The explained variances are the R^2^s of the zero-or-one-inflated beta regression models fitted with the abundance weighted traits (AWT) of the temperature preference grouping feature as response and the AWT of the remaining grouping features separately as predictor variables (**Table D**).
(PDF)
