# Supplementary material for: Large Scale Relationship between Aquatic Insect Traits and Climate
Source: PLoS One. 2015 Jun 16;10(6):e0130025. doi: 10.1371/journal.pone.0130025 (PMC4469582; doi:10.1371/journal.pone.0130025)
Supplement: S1 File — Annual averaged abundance weighted traits across 4,752 stream sites in Germany for each order. The figure captions, sub-captions and panel captions indicate the names of orders, grouping features and traits, respectively. The gray dots indicate zero abundance, i.e. trait absence (Fig A). Extracted 35 global bioclimatic indices within the border of Germany. The indices are grouped according to their value ranges and units (°C, mm, W m-2 and no unit). The panel captions indicate the IDs of the indices (Bio_ID). Details on the indices and their IDs and units can be found in Table 2 and https://www.climond.org/Resources.aspx (Fig B). Altitudes from the mean sea level (m) within the border of Germany. Details can be found in http://asterweb.jpl.nasa.gov/gdem.asp (Fig C). Bioclimatic indices (BIs) raster cells that are covered (72%) by the bio-monitoring steam sites (Fig D). Observed multicollinearity among the 35 bioclimatic indices (BIs). Statistically significant (p<0.001) pairwise correlation coefficients (Pearson) are reported with scatterplots and histograms showing distribution. Details on the indices and their IDs and units can be found in Table 2 and https://www.climond.org/Resources.aspx (Fig E). Steps of the trait-climate spatial relationship analysis (Fig F). (PDF) [file pone.0130025.s001.pdf]

S1 File. Supporting figures

Large scale relationship between aquatic insect traits and climate

Avit Kumar Bhowmik[1*], Ralf B. Schäfer[1]

[1] Quantitative Landscape Ecology, Institute for Environmental Sciences
University of Koblenz-Landau, D-76829 Landau in der Pfalz, Germany

* Corresponding author

E-mail: bhowmik@uni-landau.de

S1 File. Supporting figures. Annual averaged abundance weighted traits across 4,752 stream sites in Germany for each order. The figure captions, sub-captions and panel captions indicate the names of orders, grouping features and traits, respectively. The gray dots indicate zero abundance, i.e. trait absence (**Figure A**). Extracted 35 global bioclimatic indices within the border of Germany. The indices are grouped according to their value ranges and units (^{0}C, mm, W m^{-2} and no unit). The panel captions indicate the IDs of the indices (Bio_ID). Details on the indices and their IDs and units can be found in Table 2 and **https://www.climond.org/Resources.aspx** (**Figure B**). Altitudes from the mean sea level (m) within the border of Germany. Details can be found in **http://asterweb.jpl.nasa.gov/gdem.asp** (**Figure C**). Bioclimatic indices (BIs) raster cells that are covered (72 %) by the bio-monitoring steam sites (**Figure D**). Observed multicollinearity among the 35 bioclimatic indices (BIs). Statistically significant ($p<0.001$) pairwise correlation coefficients (Pearson) are reported with scatterplots and histograms showing distribution. Details on the indices and their IDs and units can be found in Table 2 and **https://www.climond.org/Resources.aspx** (**Figure E**). Steps of the trait-climate spatial relationship analysis (**Figure F**).

Diptera

Biological traits

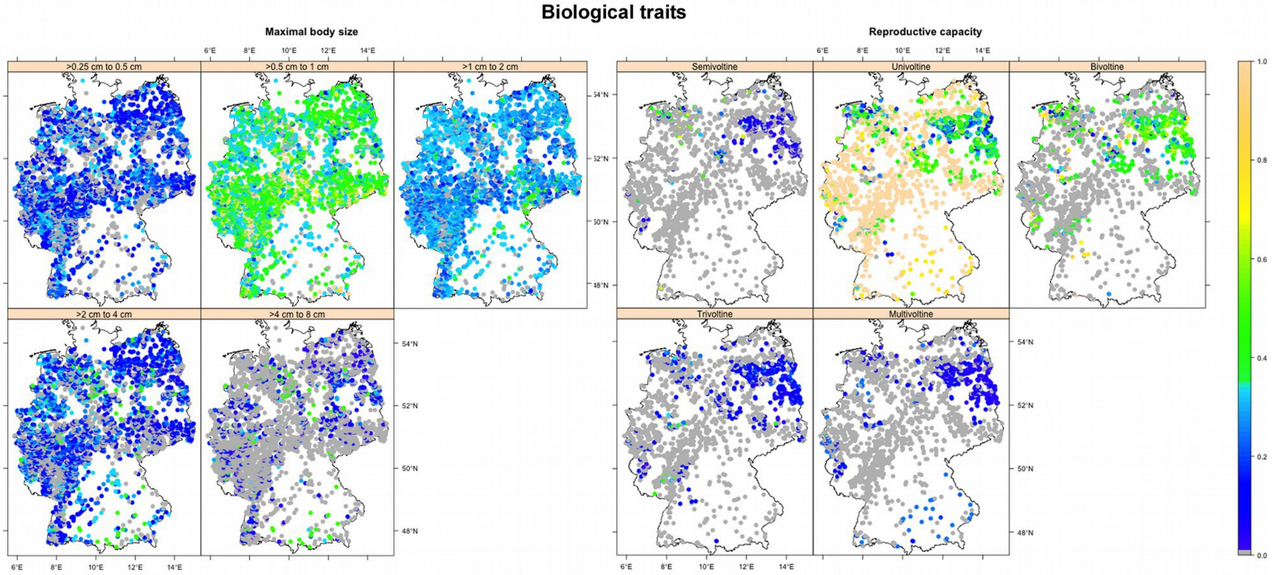

Ecological traits

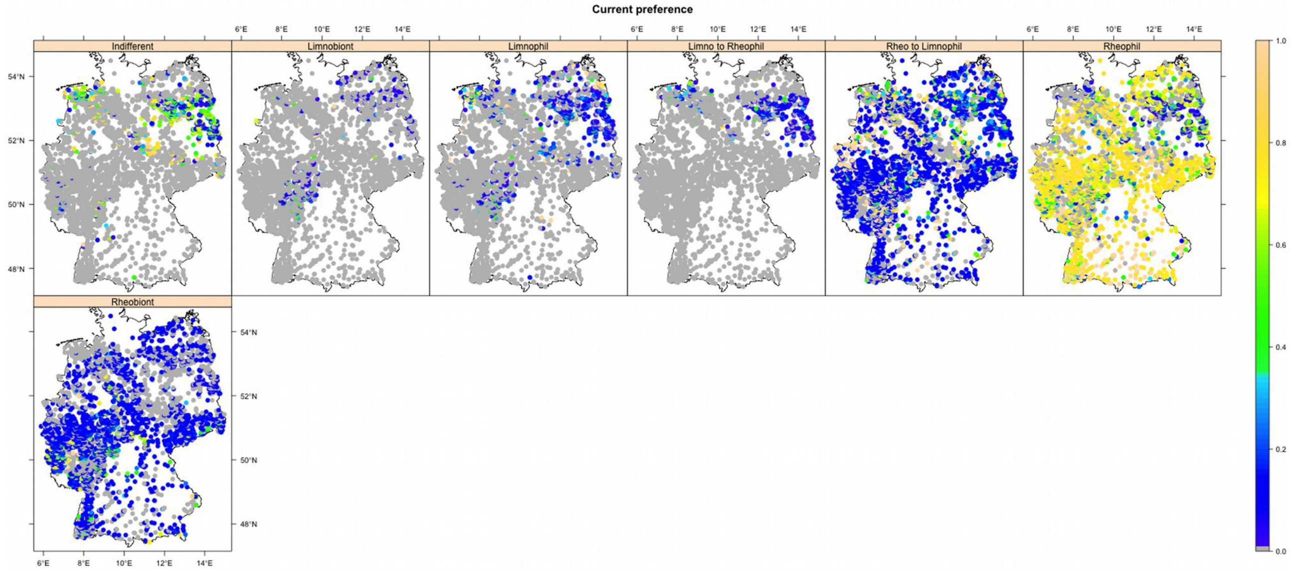

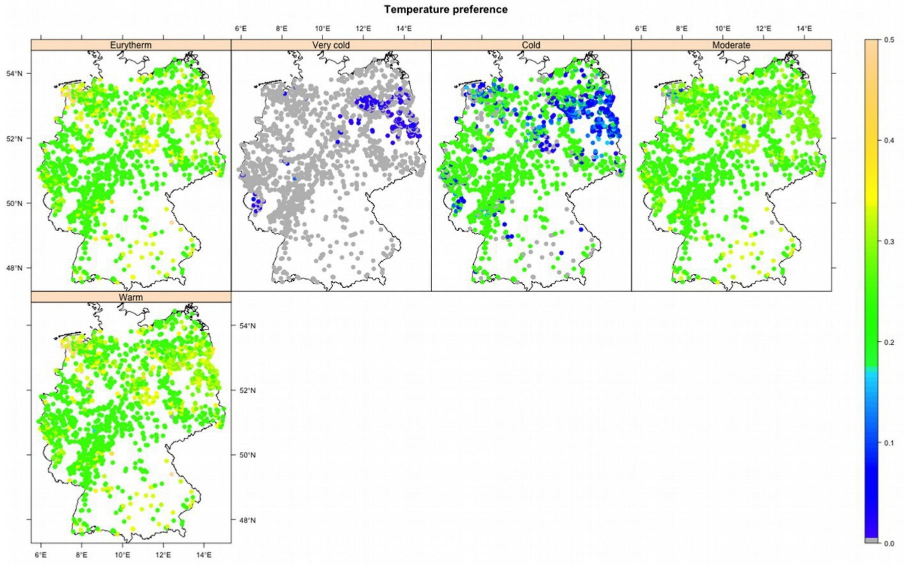

(Continued to next page)

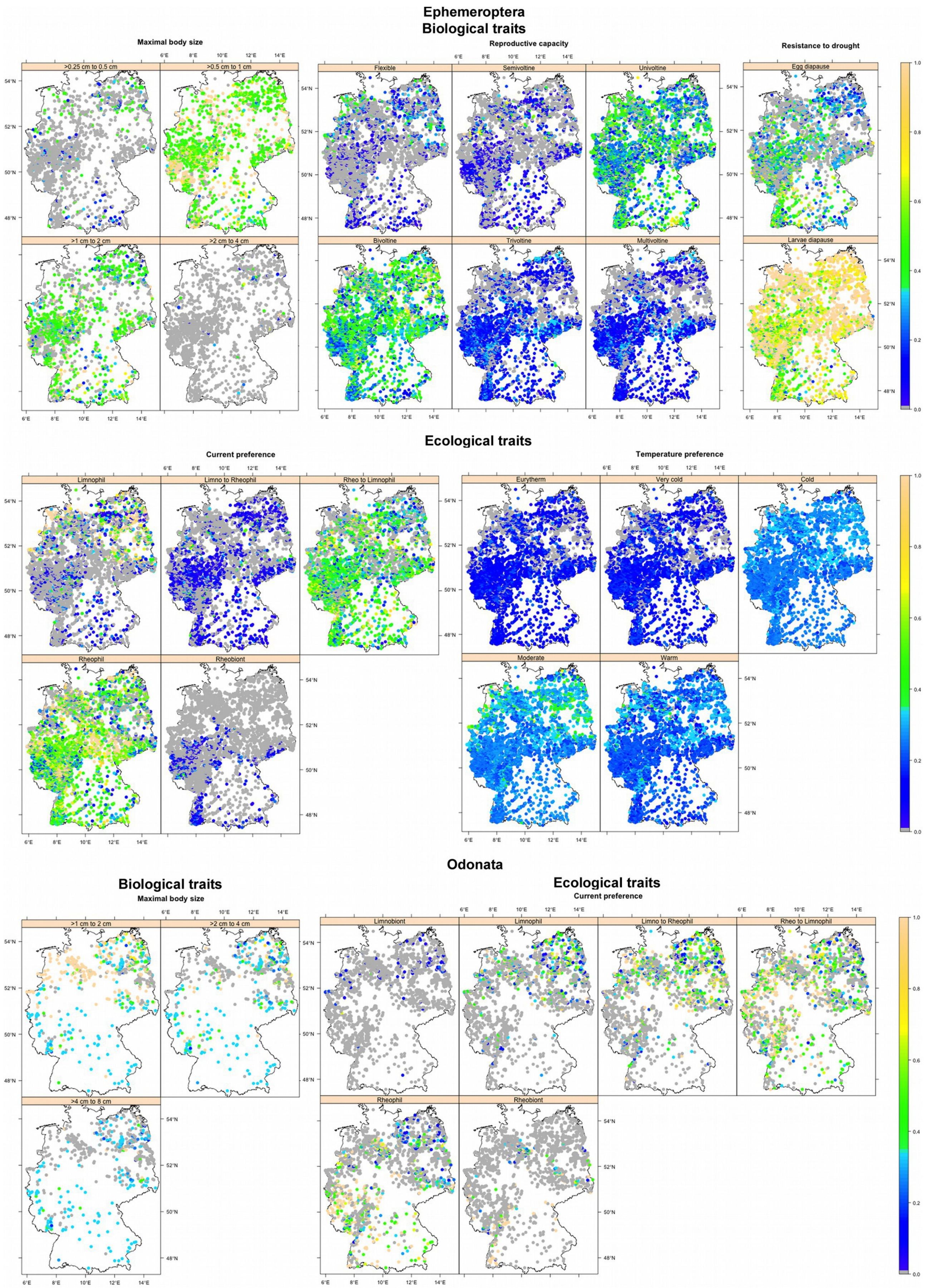

(Continued to next page)

Plecoptera
Biological traits

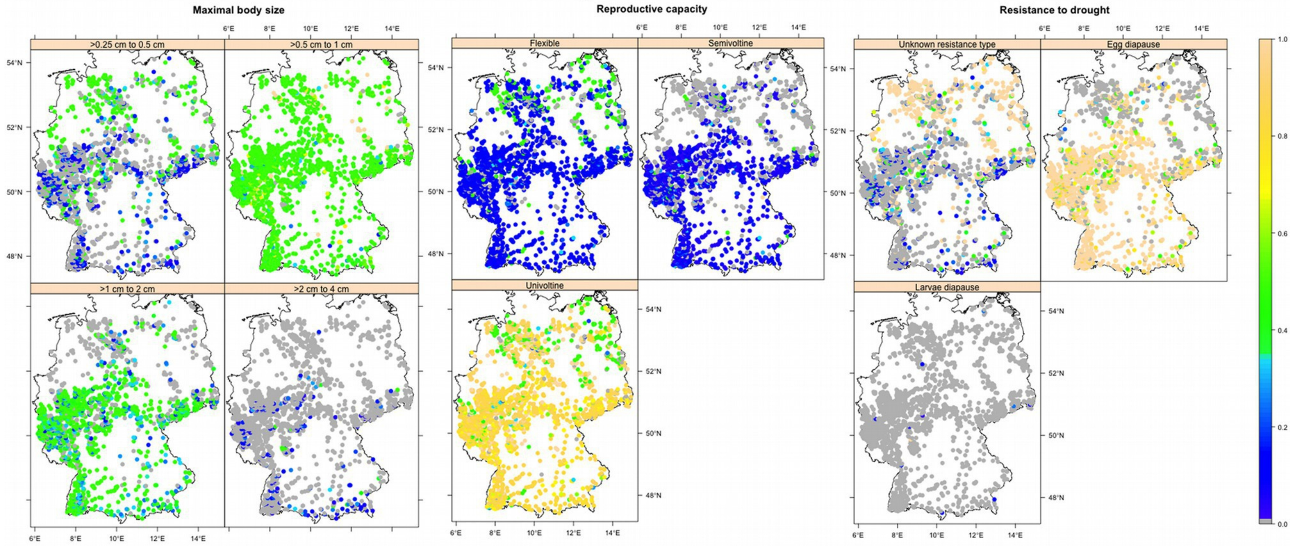

Ecological traits

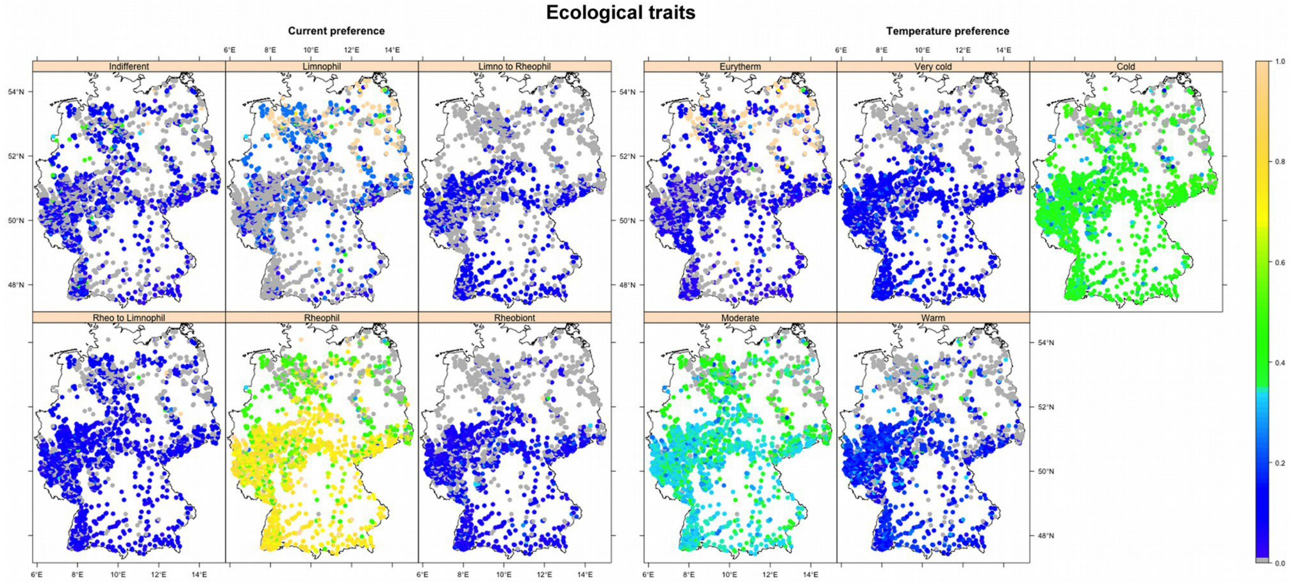

Trichoptera
Biological traits

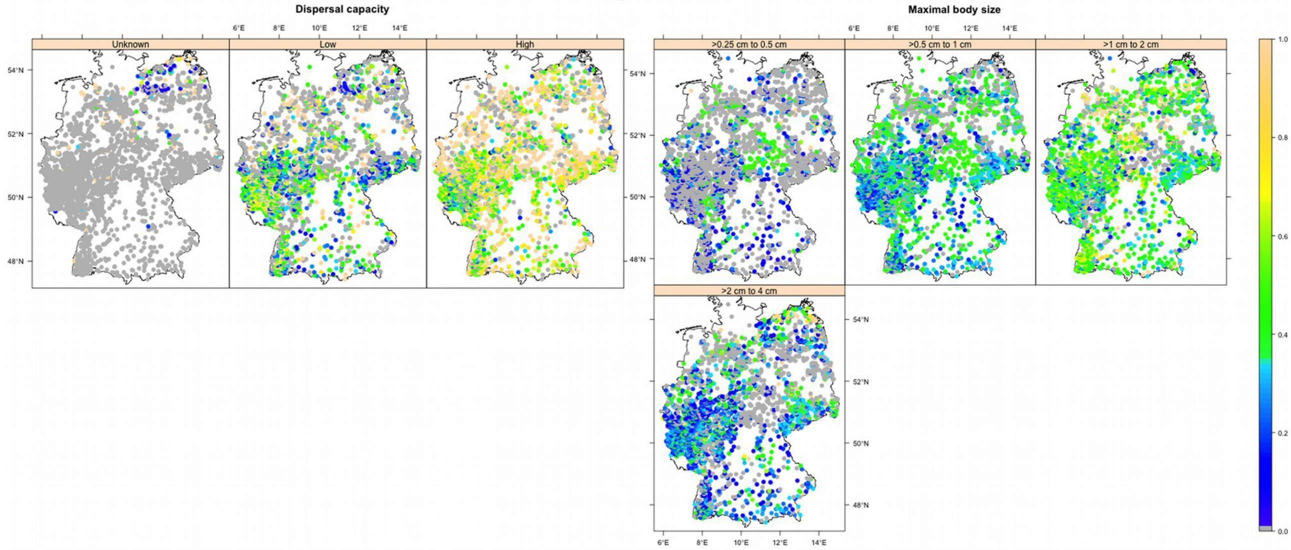

(Continued to next page)

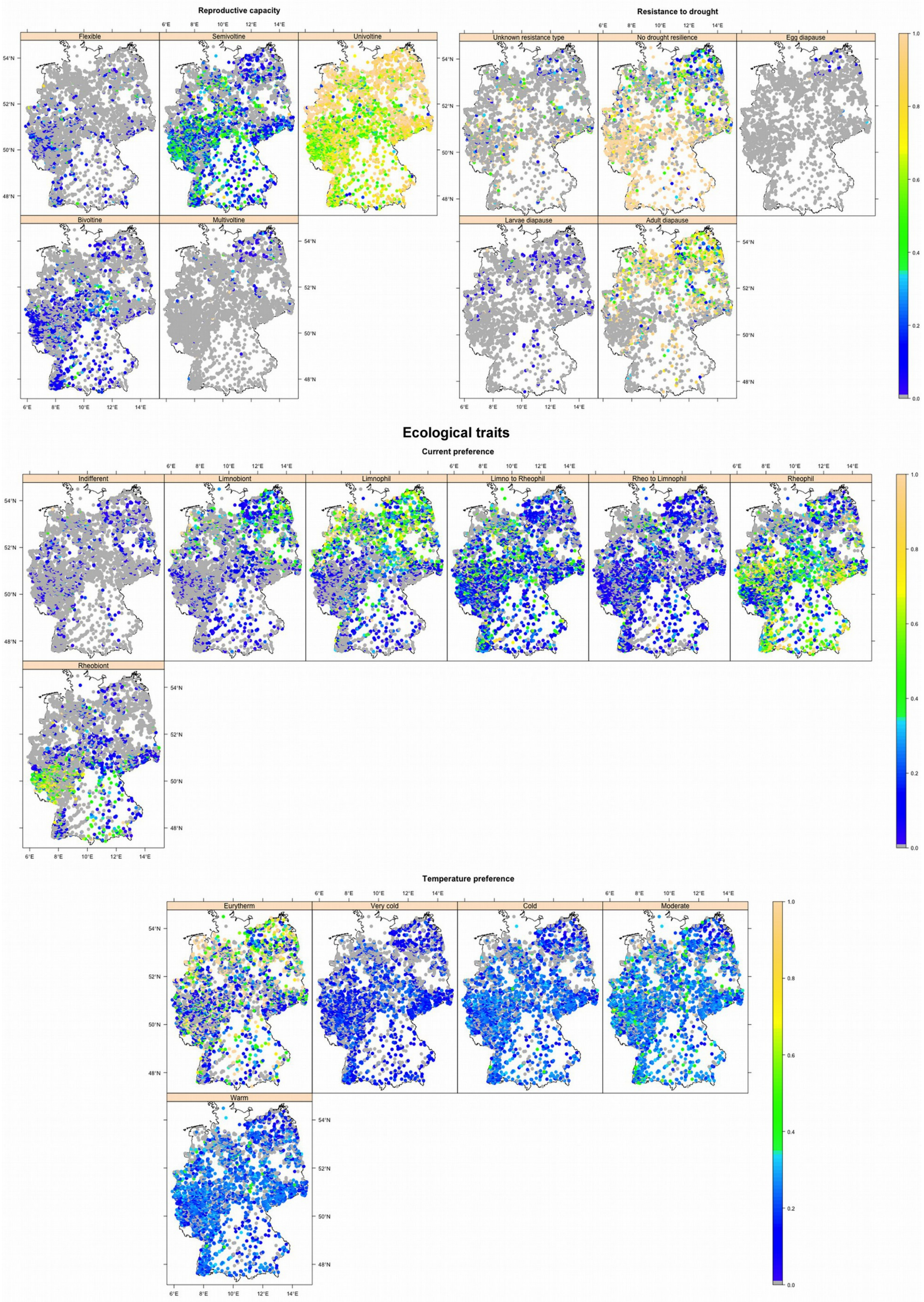

Figure A. Annual averaged abundance weighted traits across 4,752 stream sites in Germany for each order. The figure captions, sub-captions and panel captions indicate the names of orders, grouping features and traits, respectively. The gray dots indicate zero abundance, i.e. trait absence.

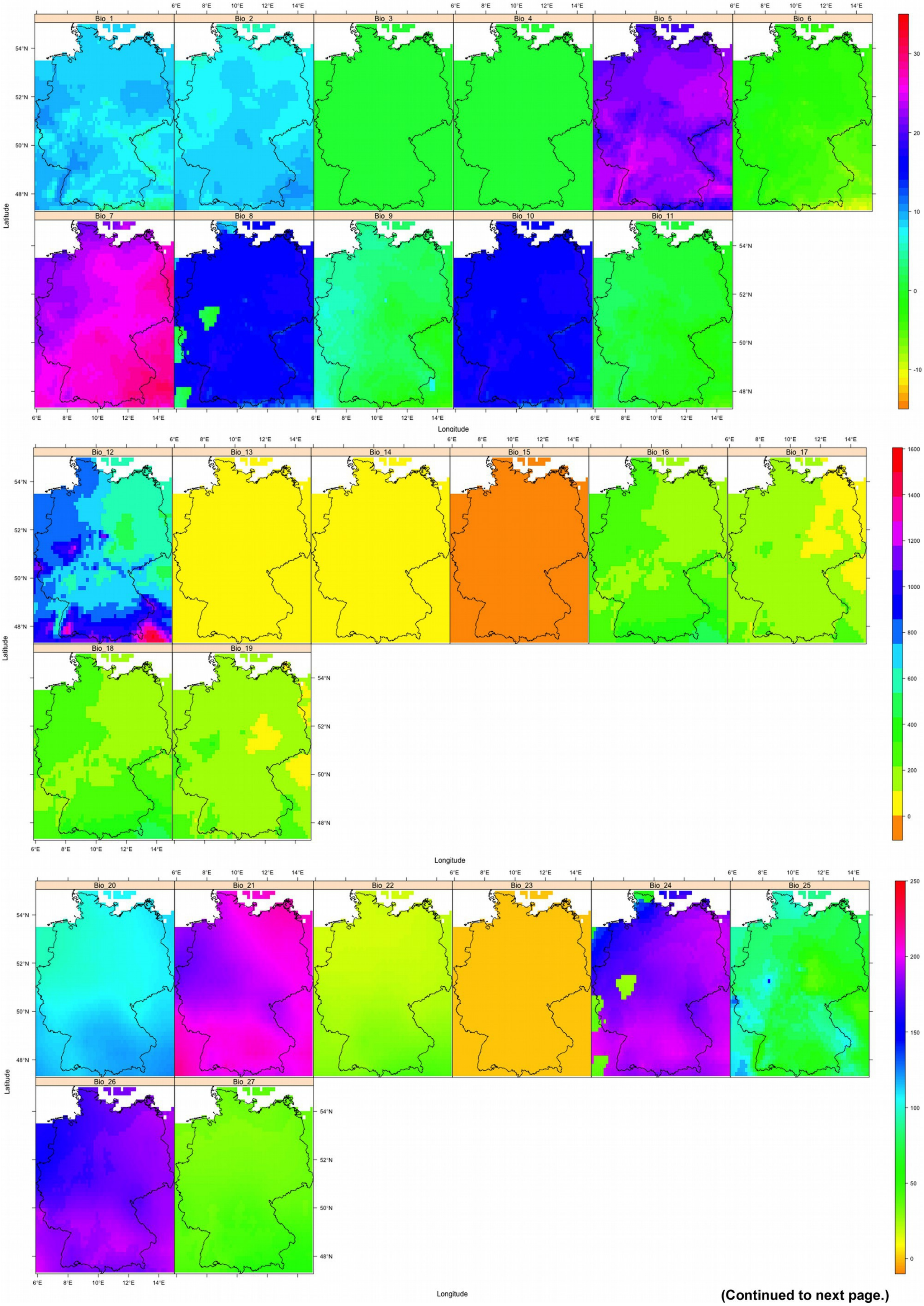

(Continued to next page.)

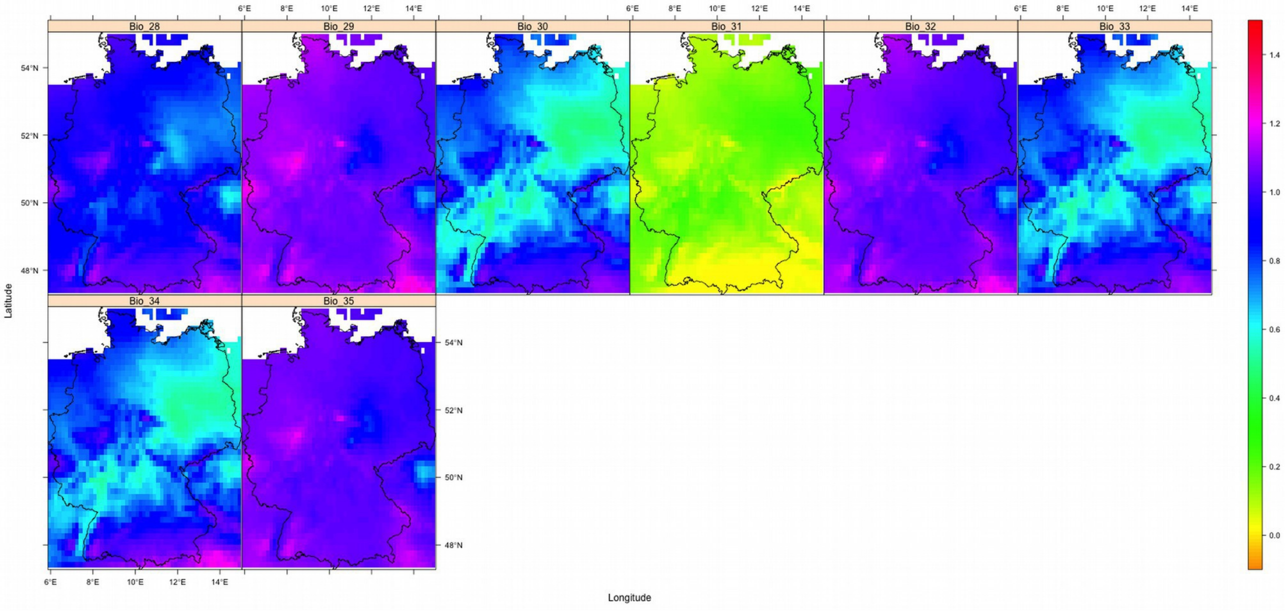

Figure B. Extracted 35 global bioclimatic indices within the border of Germany. The indices are grouped according to their value ranges and units (^{0}C, mm, W m^{-2} and no unit). The panel captions indicate the IDs of the indices (Bio_ID). Details on the indices and their IDs and units can be found in Table 2 and **https://www.climond.org/Resources.aspx**.

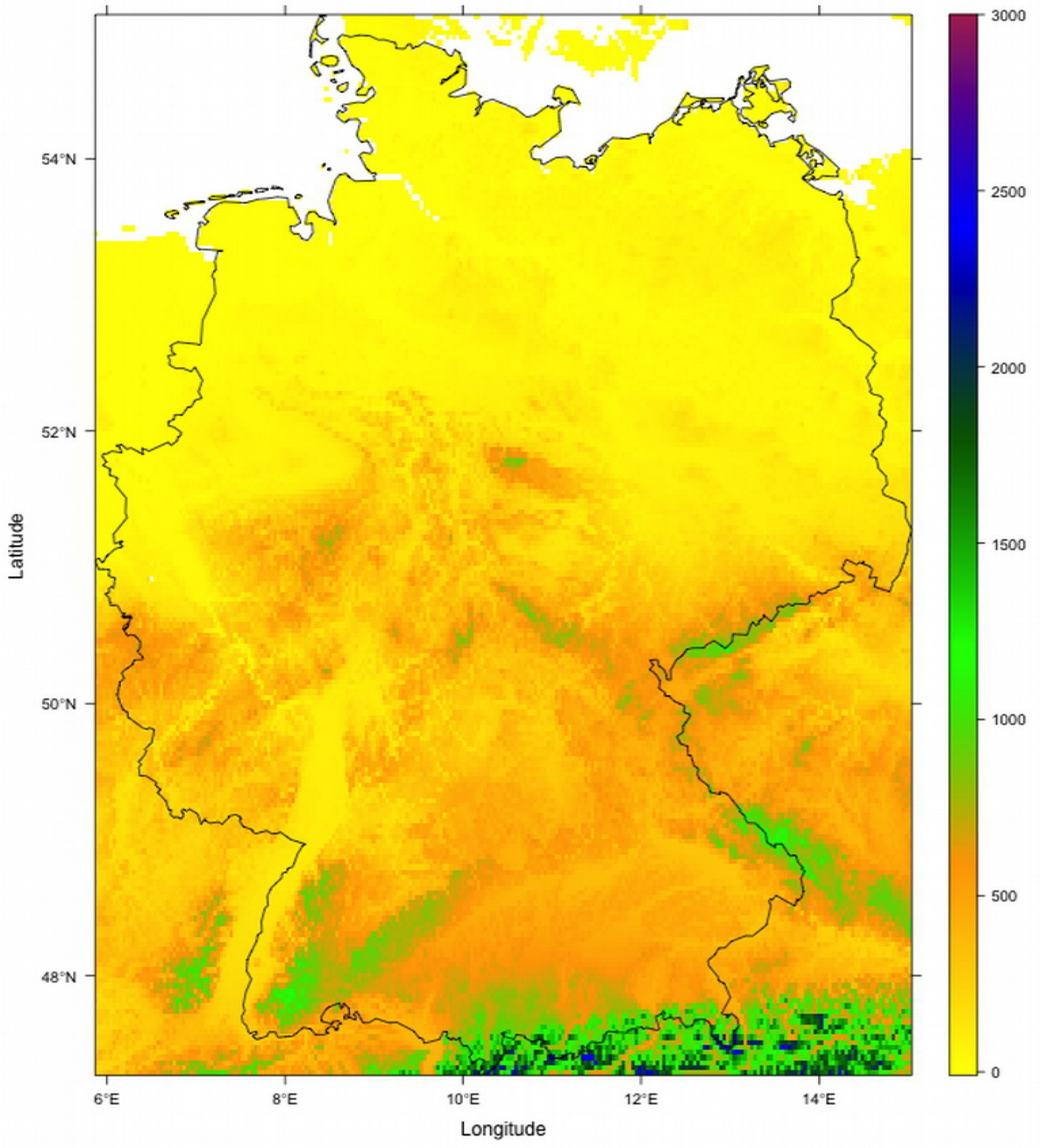

Figure C. Altitudes from the mean sea level (m) within the border of Germany. Details can be found in **http://asterweb.jpl.nasa.gov/gdem.asp**.

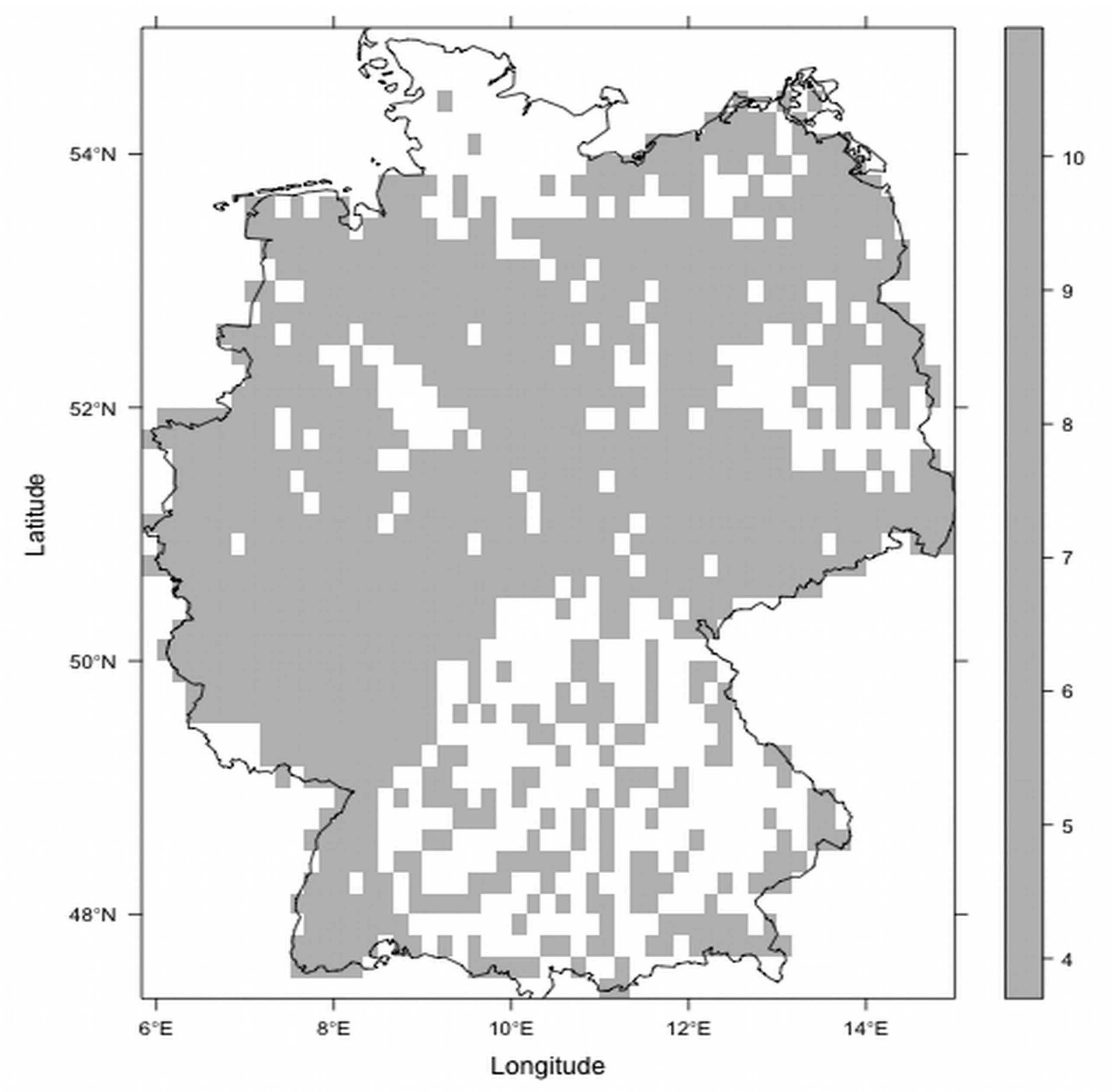

Figure D. Bioclimatic indices (BIs) raster cells that are covered (72 %) by the bio-monitoring steam sites.

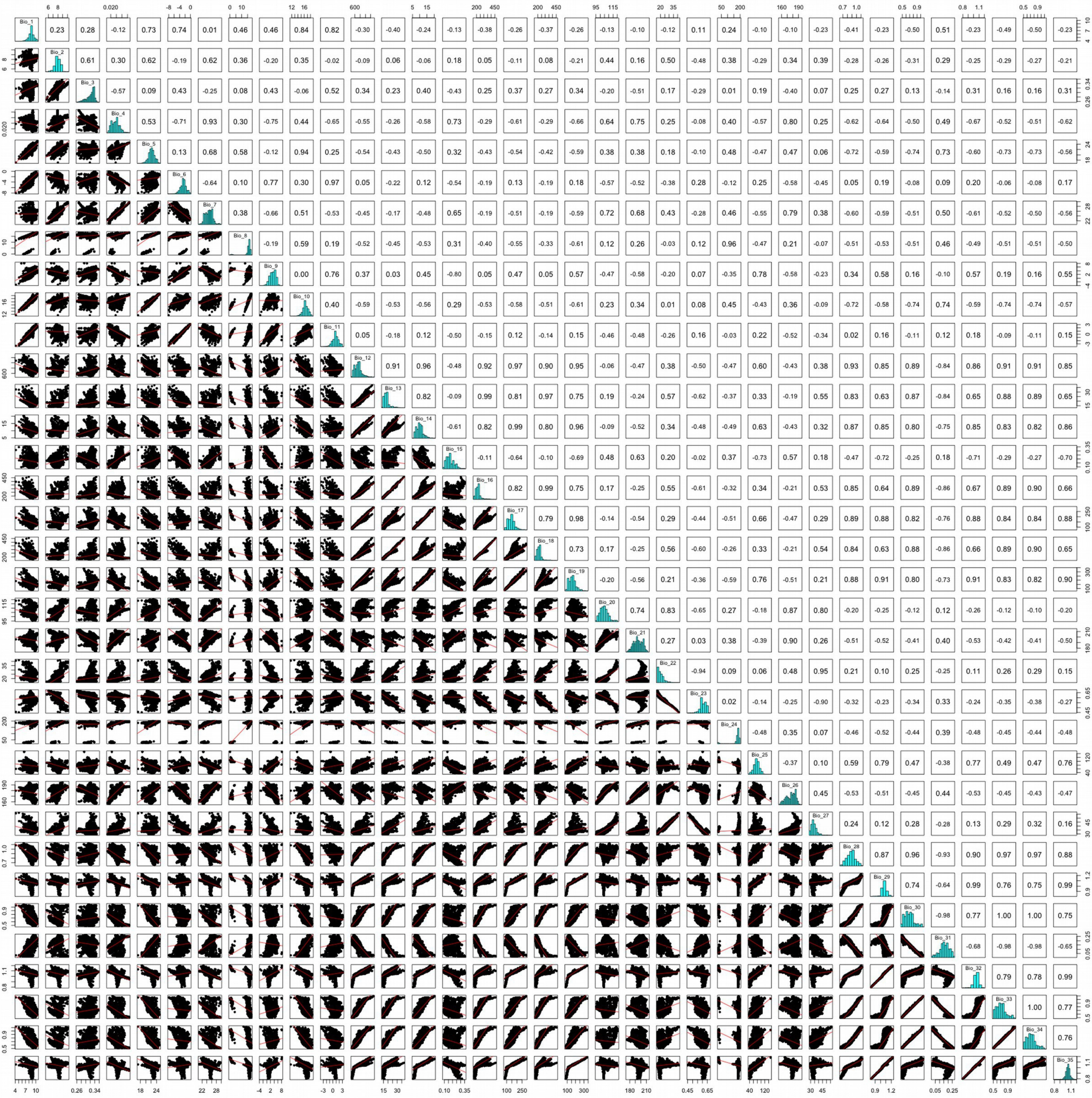

Figure E. Observed multicollinearity among the 35 bioclimatic indices (BIs). Statistically significant (p<0.001) pairwise correlation coefficients (Pearson) are reported with scatterplots and histograms showing distribution. Details on the indices and their IDs and units can be found in Table 2 and https://www.climond.org/Resources.aspx.

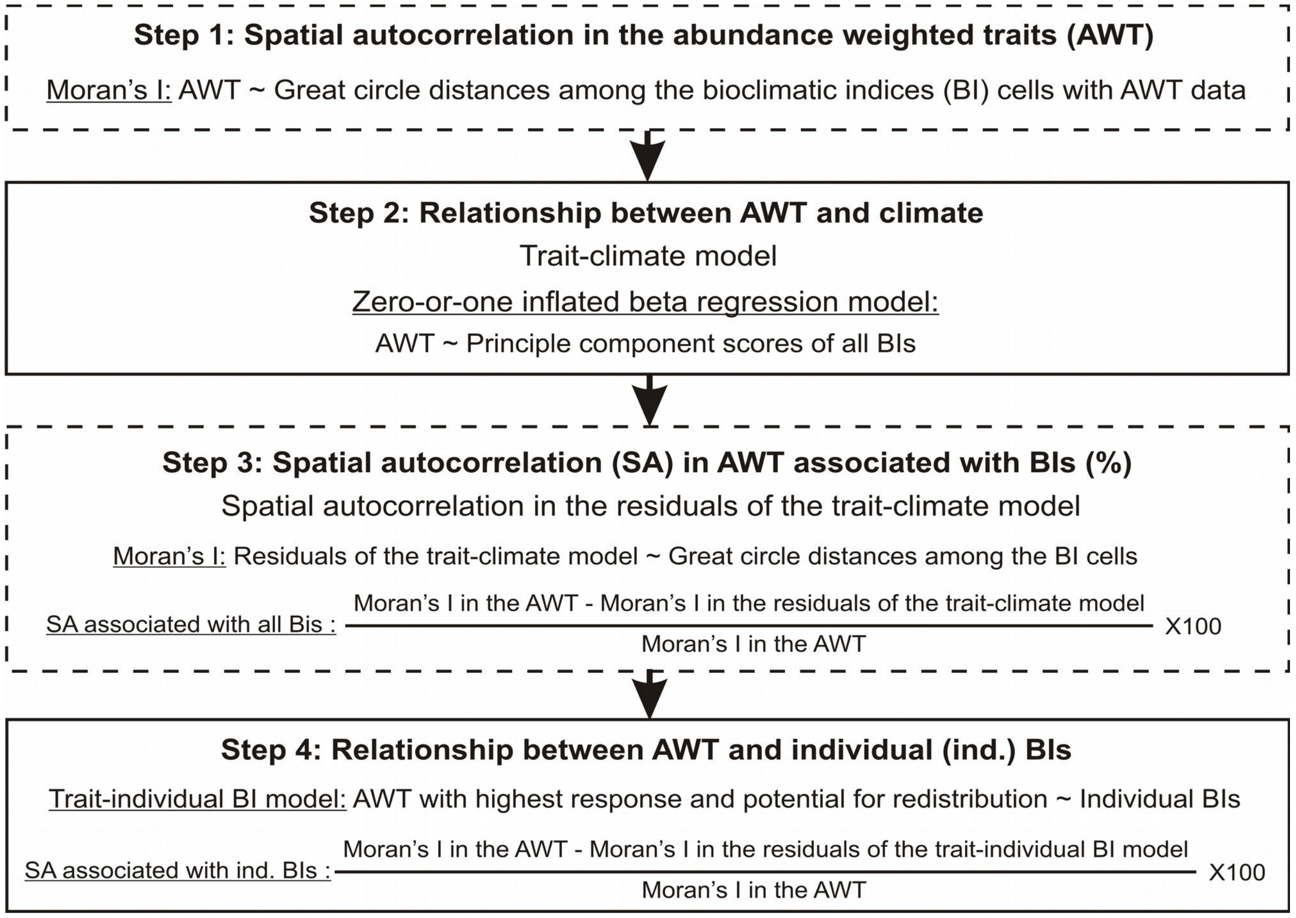

Figure F. Steps of the trait-climate spatial relationship analysis.
