# Supplementary material for: Large Scale Relationship between Aquatic Insect Traits and Climate
Source: PLoS One. 2015 Jun 16;10(6):e0130025. doi: 10.1371/journal.pone.0130025 (PMC4469582; doi:10.1371/journal.pone.0130025)
Supplement: S2 File — Membership states of the five insect orders (%) for the traits of each grouping feature. The membership state (%) of an order for a trait was computed as the median of the membership states of all taxa in that order for that trait. The membership states were then scaled by the total of the membership states of an order for the traits of a grouping feature so that the membership states sum to 100% for each grouping feature (Table A). Spatial autocorrelations (Moran's I values) and gradients (Pearson correlations with longitude, latitude and altitude) for the bioclimatic indices (BIs) extracted at the stream sites. The Moran's I values and Pearson correlation coefficients are statistically significant at p<0.001. Details on the indices and their IDs and units can be found in Table 2 and https://www.climond.org/Resources.aspx (Table B). Spatial autocorrelations (global Moran's I) for abundance weighted traits in each stream macroinvertebrate order and in the full data. Observed Moran's I values are statistically significant at p<0.001 (Table C). Relationship between the traits of temperature preference grouping feature and the traits of remaining grouping features in terms of explained variance (%). The explained variances are the R2s of the zero-or-one-inflated beta regression models fitted with the abundance weighted traits (AWT) of the temperature preference grouping feature as response and the AWT of the remaining grouping features separately as predictor variables (Table D). (PDF) [file pone.0130025.s002.pdf]

S2 File. Supporting tables

Large scale relationship between aquatic insect traits and climate

Avit Kumar Bhowmik[1*], Ralf B. Schäfer[1]

[1] Quantitative Landscape Ecology, Institute for Environmental Sciences
University of Koblenz-Landau, D-76829 Landau in der Pfalz, Germany

* Corresponding author

E-mail: bhowmik@uni-landau.de

S2 File. Supporting tables. Membership states of the five insect orders (%) for the traits of each grouping feature. The membership state (%) of an order for a trait was computed as the median of the membership states of all taxa in that order for that trait. The membership states were then scaled by the total of the membership states of an order for the traits of a grouping feature so that the membership states sum to 100 % for each grouping feature (**Table A**). Spatial autocorrelations (Moran's I values) and gradients (Pearson correlations with longitude, latitude and altitude) for the bioclimatic indices (BIs) extracted at the stream sites. The Moran's I values and Pearson correlation coefficients are statistically significant at $p<0.001$. Details on the indices and their IDs and units can be found in Table 2 and https://www.climond.org/Resources.aspx (**Table B**). Spatial autocorrelations (global Moran's I) for abundance weighted traits in each stream macroinvertebrate order and in the full data. Observed Moran's I values are statistically significant at $p<0.001$ (**Table C**). Relationship between the traits of temperature preference grouping feature and the traits of remaining grouping features in terms of explained variance (%). The explained variances are the R^2s of the zero-or-one-inflated beta regression models fitted with the abundance weighted traits (AWT) of the temperature preference grouping feature as response and the AWT of the remaining grouping features separately as predictor variables (**Table D**).

Table A. Membership states of the five insect orders (%) for the traits of each grouping feature. The membership state (%) of an order for a trait was computed as the median of the membership states of all taxa in that order for that trait. The membership states were then scaled by the total of the membership states of an order for the traits of a grouping feature so that the membership states sum to 100 % for each grouping feature.

**Grouping features and traits**	**Aquatic insect orders**				
	**Diptera**	**Ephemeroptera**	**Odonata**	**Plecoptera**	**Trichoptera**
**Biological traits**					
Dispersal capacity					
Unknown	NA	NA	NA	NA	9
Low	NA	100*	NA	100*	36
High	NA	NA	NA	NA	55*
Maximal body size					
> 0.25 cm to 0.5 cm	11	3	NA	6	11
> 0.5 cm to 1 cm	39*	43	3	39*	37
> 1 cm to 2 cm	29	50*	44*	36	39*
> 2 cm to 4 cm	14	3	44*	19	13
> 4 cm to 8 cm	7	NA	9	NA	NA
Reproductive capacity					
Flexible	NA	4	NA	7	5
Semivoltine	4	2	NA	15	10
Univoltine	39	64*	NA	79*	80*
Bivoltine	44*	23	NA	NA	4
Trivoltine	11	5	NA	NA	NA
Multivoltine	3	1	NA	NA	1
Resistance to drought					
Unknown resistance type	NA	NA	NA	28	4
No drought resilience	NA	NA	NA	NA	49*
Egg diapause	NA	60*	NA	62*	3
Larvae diapause	NA	40	NA	10	4
Adult diapause	NA	NA	NA	NA	40
**Ecological traits**					
Current preference					
Indifferent	13	NA	NA	3	3
Limnobiont	7	1	21	NA	19
Limnophil	17	11	49*	1	16
Limno to Rheophil	4	10	14	2	12
Rheo to Limnophil	14	27	7	10	13

Rheophil	31*	45*	7	80*	26*
Rheobiont	13	7	3	5	11
Temperature preference					
Eurytherm	30*	7	NA	1	48*
Very cold	3	15	NA	16	6
Cold	14	27	NA	46*	14
Moderate	26	32*	NA	27	23
Warm	27	19	NA	10	10

[NA] Trait not occurring
* The highest membership state (%) of an insect order for a trait in a grouping feature

Table B. Spatial autocorrelations (Moran's I values) and gradients (Pearson correlations with longitude, latitude and altitude) for the bioclimatic indices (BIs) extracted at the stream sites. The Moran's I values and Pearson correlation coefficients are statistically significant at $p<0.001$. Details on the indices and their IDs and units can be found in Table 2 and https://www.climond.org/Resources.aspx.

Bioclimatic indices (BIs)	Spatial autocorrelation (Global Moran's I)	Correlation (Pearson) with spatial variables		
		Longitude	Latitude	Altitude
Bio01	0.18	-0.42	0.30	-0.77
Bio02	0.29	0.16	-0.77	0.52
Bio03	0.28	-0.54	-0.53	0.26
Bio04	0.36	0.86	-0.34	0.30
Bio05	0.13	0.10	-0.20	-0.32
Bio06	0.32	-0.68	0.49	-0.76
Bio07	0.34	0.68	-0.58	0.45
Bio08	0.09	0.28	0.13	-0.3
Bio09	0.30	-0.75	0.33	-0.58
Bio10	0.12	0.00	0.15	-0.64
Bio11	0.30	-0.70	0.40	-0.73
Bio12	0.27	-0.27	-0.58	0.69
Bio13	0.30	0.04	-0.67	0.80
Bio14	0.26	-0.45	-0.55	0.60
Bio15	0.30	0.72	-0.28	0.33
Bio16	0.30	0.03	-0.66	0.79
Bio17	0.25	-0.44	-0.51	0.59
Bio18	0.30	0.05	-0.66	0.79
Bio19	0.24	-0.53	-0.39	0.47
Bio20	0.38	0.36	-0.78	0.68
Bio21	0.29	0.43	-0.23	0.11
Bio22	0.40	0.16	-0.89	0.84
Bio23	0.41	-0.09	0.92	-0.86
Bio24	0.13	0.43	-0.14	0.08
Bio25	0.20	-0.53	0.02	0.04
Bio26	0.33	0.53	-0.51	0.35
Bio27	0.37	0.25	-0.78	0.83
Bio28	0.24	-0.33	-0.37	0.56
Bio29	0.19	-0.48	-0.13	0.29
Bio30	0.25	-0.09	-0.46	0.67
Bio31	0.26	0.06	0.47	-0.64

Bio32	0.20	-0.51	-0.16	0.31
Bio33	0.25	-0.14	-0.47	0.66
Bio34	0.25	-0.12	-0.48	0.69
Bio35	0.20	-0.51	-0.21	0.30

Table C. Spatial autocorrelations (global Moran's I) for abundance weighted traits in each stream macroinvertebrate order and in the full data. Observed Moran's I values are statistically significant at p<0.001.

Grouping features and traits	Aquatic insect orders					
	Diptera	Ephemeroptera	Odonata	Plecoptera	Trichoptera	Full data
**Biological traits**						
Dispersal capacity						
Unknown	NA	NA	NA	NA	0.05	0.03
Low	NA	*	NA	*	0.03	0.04
High	NA	NA	NA	NA	0.02	0.03
Average	*NA*	*NA*	*NA*	*NA*	*0.03*	*0.03*
Maximal body size						
> 0.25 cm to 0.5 cm	0.02	0.04	NA	0.12	0.03	0.04
> 0.5 cm to 1 cm	0.03	0.02	NA	0.03	0.03	0.03
> 1 cm to 2 cm	0.02	0.04	0.13	0.15	0.03	0.03
> 2 cm to 4 cm	0.04	0.03	0.06	0.04	0.03	0.03
> 4 cm to 8 cm	0.02	NA	0.08	NA	NA	0.04
Average	*0.03*	*0.03*	*0.09*	*0.09*	*0.03*	*0.03*
Reproductive capacity						
Flexible	NA	0.07	NA	0.08	0.02	0.03
Semivoltine	0.03	0.03	NA	0.03	0.08	0.02
Univoltine	0.12	0.03	NA	0.04	0.07	0.04
Bivoltine	0.12	0.03	NA	NA	0.04	0.05
Trivoltine	0.06	0.10	NA	NA	NA	0.04
Multivoltine	0.05	0.08	NA	NA	0.01	0.03
Average	*0.08*	*0.06*	*NA*	*0.05*	*0.04*	*0.04*
Resistance to drought						
Unknown resistance type	NA	NA	NA	0.19	0.02	0.03
No drought resilience	NA	NA	NA	NA	0.10	0.04
Egg diapause	NA	0.08	NA	0.2	0.01	0.12
Larvae diapause	NA	0.08	NA	0.00	0.01	0.17
Adult diapause	NA	NA	NA	NA	0.13	0.02
Average	*NA*	*0.08*	*NA*	*0.13*	*0.05*	*0.08*
**Ecological traits**						
Current preference						
Indifferent	0.10	NA	NA	0.03	0.02	0.08
Limnobiont	0.01	NA	0.02	NA	0.10	0.07

Limnophil	0.05	0.13	0.08	0.17	0.16	0.14
Limno to Rheophil	0.07	0.01	0.10	0.02	0.04	0.02
Rheo to Limnophil	0.04	0.07	0.04	0.00	0.01	0.03
Rheophil	0.09	0.05	0.11	0.20	0.11	0.13
Rheobiont	0.06	0.07	0.05	0.06	0.13	0.15
Average	*0.06*	*0.07*	*0.07*	*0.08*	*0.08*	*0.09*
Temperature preference						
Eurytherm	0.08	0.08	NA	0.15	0.05	0.10
Very cold	0.06	0.11	NA	0.14	0.06	0.15
Cold	0.08	0.03	NA	0.10	0.07	0.13
Moderate	0.03	0.09	NA	0.08	0.01	0.01
Warm	0.07	0.03	NA	0.09	0.05	0.06
Average	*0.06*	*0.07*	*NA*	*0.11*	*0.05*	*0.09*
Average over traits and orders	***0.06***	***0.06***	***0.07***	***0.09***	***0.05***	***0.06***

[NA] Trait not occurring
* Trait omitted from the analysis because of zero variability (i.e. all taxa have same trait) and therefore the abundance weighted trait cannot be computed

Table D. Relationship between the traits of temperature preference grouping feature and the traits of remaining grouping features in terms of explained variance (%). The explained variances are the R^2s of the zero-or-one-inflated beta regression models fitted with the abundance weighted traits (AWT) of the temperature preference grouping feature as response and the AWT of the remaining grouping features separately as predictor variables.

Remaining grouping features and traits	Traits of temperature preference grouping feature				
	**Eurytherm**	**Very cold**	**Cold**	**Moderate**	**Warm**
**Biological traits**					
Dispersal capacity					
Unknown	3.6*	0.1	2.0	0.0	0.0
Low	3.2	1.2*	9.8*	1.5*	15
High	0.9	0.5	4.5	1.4	17*
Maximal body size					
> 0.25 cm to 0.5cm	2.0	10	2.8	3.4*	2.1*
> 0.5 cm to 1 cm	1.9	0.0	1.8	0.4	0.6
> 1 cm to 2 cm	4.4	10	4.3	0.2	1.7
> 2 cm to 4 cm	5.7*	0.0	2.7	1.1	0.1
> 4 cm to 8 cm	3.2	13*	5.8*	0.3	1.9
Reproductive capacity					
Flexible	1.6	3.2	1.1	9.7	3.2
Semivoltine	0.1	9.0	16*	1.7	16
Univoltine	2.9	15*	7.5	4.2	6.7
Bivoltine	2.7	1.0	4.5	0.0	0.8
Trivoltine	0.7	7.0	1.3	10	0.8
Multivoltine	1.7	15	0.1	14*	36*
Resistance to drought					
Unknown resistance type	1.8	6.2	0.3	1.8	11
No drought resilience	6.7	2.8	6.3	0.0	0.3
Egg diapause	28*	13	30*	1.4	6.6
Larvae diapause	0.0	25*	0.7	0.0	0.6
Adult diapause	15	0.0	16	3.7*	14*
**Ecological traits**					
Current preference					
Indifferent	13	36	30	0.9	12*
Limnobiont	12	14	16	0.6	0.8
Limnophil	18	41	27	1.3*	4.6
Limno to Rheophil	0.5	1.6	0.7	0.1	0.0
Rheo to Limnophil	3.0	0.0	0.0	0.6	0.0

Rheophil	26*	42*	35*	0.2	2.6
Rheobiont	5.3	16	12	0.8	6.6

* The highest explained variance in the traits of the temperature preference grouping feature by the traits of the remaining grouping features
